# UPLC-MS/MS determination of 71 neuropsychotropic drugs in human serum

**DOI:** 10.1016/j.heliyon.2024.e32274

**Published:** 2024-06-10

**Authors:** Weifeng Jin, Jianhua Wang, Shuzi Chen, Qing Chen, Dan Li, Mengyuan Zhu, Xiaomei Fu, Yingyu Huang, Ping Lin

**Affiliations:** a Department of Medical Laboratory, Shanghai Mental Health Center, Shanghai Jiao Tong University School of Medicine, China; b Shanghai Biotree Biomedical Technology Co, China

**Keywords:** UPLC-MS/MS, TDM, Neuropsychotropic drugs

## Abstract

In this study, a UPLC-MS/MS method was developed for the rapid detection of 71 neuropsychotropic drugs in human serum for drug concentration monitoring and toxicity screening. The analytes were separated from the biological matrix by protein precipitation using a methanol-acetonitrile solvent mixture. The chromatographic separation was performed on a Kromasil ClassicShell C18 column (2.1*50 mm, 2.5 μ m) with gradient elution using acetonitrile-0.2 % acetic acid and 10 mM ammonium acetate as the mobile phases (flow rate 0.4 mL/min, column temperature 40 °C, injection volume 5 μL). An electrospray ion source in both positive and negative ion modes with multiple ion monitoring was used. The total run time was 6 min. All compounds were quantified using the isotope internal standard method. Totally, 71 drugs were detected within their linear ranges with correlation coefficients greater than 0.990. The intra- and inter-batch precision relative standard deviations (RSDs) for the low, medium, and high concentration points were less than 15 %, with an accuracy of 90%–110 %. All compounds except Moclobemide N-oxindole are stabilised within 7 days. The relative matrix effect results for each analyte were within ±20 % of the requirements. The method is validated according to Clinical and Laboratory Standards Institute guidelines, easy to use, and has a low cost.

## 1 Introduction

Mental illnesses account for a large proportion of clinically relevant diseases and are currently treated mainly by administering neuropsychotropic drugs that offer symptomatic control, but regular monitoring of blood levels is required due to interpatient differences, narrow therapeutic window for the drugs, high toxicity, and susceptibility to adverse reactions [[1], [2], [3], [4], [5]].

Therapeutic drug monitoring (TDM) is an effective way of tailoring medication regimens to individual patients. According to the TDM expert group of the European Association of Neuropsychopharmacology and Pharmacopsychiatry (AGNP), which updated and published the consensus guidelines for psychiatric TDM in 2017, the number of drug classes was expanded to 154, and neuropsychotropic drugs were divided into various categories, including antidepressants, antipsychotics, antiepileptics, and affective stabilizers [6]. To guide and standardize TDM in medical institutions and medical consultancies, the Professional Committee on Therapeutic Drug Monitoring and Research of the Chinese Society of Pharmacology formulated the Expert Consensus on the Specification of Therapeutic Drug Monitoring Work in 2022 [7] and published the Expert Consensus on the Use of Chromatographic Techniques for Quality Assurance of Therapeutic Drug Monitoring in 2021 [8]. Several published guidelines recommend liquid chromatography-tandem mass spectrometry (LC-MS/MS) as an effective way to monitor drug concentrations in the blood.

LC-MS/MS methods can detect neuropsychotropic drugs with high sensitivity and accuracy [[9], [10], [11]]. These methods for detecting single or multiple classes of neuropsychotropic drugs by MS have been reported, but these methods have the following shortcomings: 1) Low throughput: most existing methods are designed for the simultaneous determination of multiple drugs of a single class [12] or separate determination of individual drugs [[13], [14], [15], [16], [17]]. 2) Low efficiency: various methodologies have to be used to determine drugs of distinct classes, necessitating frequent alteration of columns and mobile phases during the assay. This entails increased allocation of labor, resources, and finances. Furthermore, the capacity to concurrently detect multiple drugs remains restricted, thereby reducing assay efficiency [9,[18], [19], [20], [21]]. 3) Low sensitivity: in the simultaneous detection of multiple drugs, some drugs cannot be accurately detected due to low detection signals, which affects the accuracy of detection. In addition, the accurate quantification of drugs at lower concentrations is more challenging when also simultaneously determining drugs that are present at higher concentrations in the sample.

We developed an LC-MS/MS method to detect the concentrations of a total of 71 drugs in human serum by protein precipitation: 12 antiepileptic drugs and their 2 metabolites, 17 antipsychotics and their 5 metabolites, and 21 antidepressants and their 14 metabolites. The drugs were all present in the range of AGNP guidelines. The proposed method is simple, has good accuracy and reproducibility, and can provide technical support for the rational use of neuropsychotropic drugs.

## 2 Experimental

### 2.1 Chemicals and reagents

The 71 drug standards and internal standards were obtained from Manhage (Shanghai, China) Biotechnology Co. Methanol and acetonitrile (Shanghai ANPEL Biotechnology Co., Ltd.; Shanghai, China), as well as ammonium acetate and acetic acid (Sigma-Aldrich Co., LLC.; MO, USA) were of chromatography grade. methanol and acetonitrile were from Shanghai ANPEL Biotechnology Co.,Ltd(Shanghai,China); ammonium acetate and acetic acid were from Sigma-Aldrich Co., LLC (MO, USA); Blank serum were from RealBioGroup Co., Ltd. (Shenzhen, China). Ultrapure water (Millipore Express ultrapure water system; Millipore, Bedford, USA) was used in the analysis.

### 2.2 LC-MS/MS conditions

LC-MS/MS detection was performed on Waters Acquity UPLC I-Class XEVO TQS (Waters Co., Milford, USA). The chromatographic conditions were as follows: a ClassicShell C18 column (2.1 × 50 mm, 2.5 μm, Kromasil) was used (Nouryon Co., Göteborg, Sweden). Mobile phase A was an aqueous solution of ammonium acetate (10 mM) containing 0.2 % acetic acid. Mobile phase B was acetonitrile. The mobile phase gradient conditions used are given in Table 1. The column temperature was 40 °C, and the injection volume was 5 μL. The MS conditions were as follows: electrospray ionization (ESI) source was used in the positive and negative ion, as well as multiple reaction monitoring (MRM) modes. The capillary voltage was 0.5 kV, desolvent gas temperature was 400 °C, desolvent gas flow was 1100 L/h, conical pore gas flow was 150 L/h, and nitrogen gas pressure was 7.0 bar. The parameters for MRM monitoring are shown in Table 2.

**Table 1:** Gradient elution conditions.

Time(min)	flow rate(mL/min)	mobile phaseA%	mobile phaseB%
0.0	0.4	90	10
0.3	0.4	90	10
3	0.4	50	50
4	0.4	5	95
5	0.4	5	95
5.01	0.4	90	10
6	0.4	90	10

**A:** aqueous solution of ammonium acetate (10 mM) containing 0.2 % acetic acid.

**B:** acetonitrile.

**Table 2:** Mass spectrometry detection parameters of 71 psychotropic substances.

compound	Parent （*m*/*z*）	Daughter （*m*/*z*）	Cone voltage（v）	Collision（v）	Mode
Oxcarbazepine	253.2	208.1	75	22	ESI+
Oxcarbazepine-13C6	259.2	214.1	75	22	ESI+
10-hydroxycarbazepine	255.1	194.5	35	40	ESI+
10-hydroxycarbazepine-13C6	261.2	200.5	35	39	ESI+
Epoxide-Carbamazepine	253.2	210.1	65	25	ESI+
Epoxide-Carbamazepine-13C6	259.2	242.1	65	17	ESI+
Carbamazepine	237.1	1694.1	80	35	ESI+
Carbamazepine-13C6	243.1	200.1	80	35	ESI+
Phenobarbital	231.0	187.9	36	10	ESI-
Phenobarbital-D5	236.0	193.0	36	10	ESI-
Primidone	219.2	119.0	50	22	ESI+
Primidone-D5	224.1	124.2	50	22	ESI+
Phenytoin	251.1	101.9	54	20	ESI-
Phenytoin-13C,15N2	254.0	210.1	54	18	ESI-
Valproic acid	143.1	143.1	15	10	ESI-
Valproic acid-D4	147.0	147.0	15	10	ESI-
Lamotrigine	256.0	211.1	44	40	ESI+
Lamotrigine-13C,15N4	261.0	214.0	18	40	ESI+
Topiramate	340.2	184.2	42	10	ESI+
Topiramate-13C6	346.2	190.2	42	14	ESI+
Levetiracetam	171.2	126.1	40	20	ESI+
Levetiracetam-d6	177.2	160.0	38	8	ESI+
Perampanel	350.1	219.0	38	45	ESI+
Perampanel-d5	355.2	220.0	38	36	ESI+
Zonisamide	213.1	132.1	24	28	ESI+
Zonisamide-13C2,15N2	216.1	135.1	26	14	ESI+
Clonazepam	316.1	270.0	30	20	ESI+
Clonazepam-d4	320.1	274.1	20	25	ESI+
9-Hydroxyrisperidone	427.3	207.1	68	40	ESI+
9-Hydroxyrisperidone-d4	431.3	211.2	68	40	ESI+
Risperidone	411.3	191.1	85	40	ESI+
Risperidone-d4	415.3	195.2	85	40	ESI+
N-desmethylolanzapine	299.2	256.1	66	22	ESI+
N-desmethylolanzapine-d8	307.2	260.8	54	24	ESI+
Olanzapine	313.2	256.1	10	20	ESI+
Olanzapine-d3	316.2	256.1	46	22	ESI+
N-desmethylclozapine	313.2	192.1	90	48	ESI+
N-desmethylclozapine-d8	321.2	192.0	90	48	ESI+
Clozapine	327.2	270.1	60	38	ESI+
Clozapine-d4	331.2	272.2	60	35	ESI+
Aripiprazole	448.3	284.6	46	32	ESI+
Aripiprazole-d8	456.3	293.2	68	32	ESI+
Dehydroaripiprazole	446.3	285.1	46	40	ESI+
Dehydroaripiprazole-d8	454.3	293.2	10	40	ESI+
Amisulpride	370.3	196.0	36	55	ESI+
Amisulpride-d5	375.6	196.0	36	55	ESI+
Perphenazine	404.3	171.2	58	22	ESI+
Perphenazine-d4	408.3	171.2	46	22	ESI+
Fluphenazine	438.3	171.1	58	24	ESI+
Fluphenazine-d8	446.3	179.2	46	26	ESI+
Haloperidol	376.2	165.1	55	22	ESI+
Haloperidol-d4	380.2	165.1	55	22	ESI+
Reducdhaloperidol	378.2	360.2	46	18	ESI+
Reducdhaloperidol-d4	382.2	364.2	42	18	ESI+
Flupenthixol	435.3	128.1	46	26	ESI+
Flupenthixol-d4	439.3	132.0	46	26	ESI+
Quetiapine	384.3	253.1	85	38	ESI+
Quetiapine-d4	388.3	253.1	85	38	ESI+
Thioridazine	371.2	126.1	78	30	ESI+
Thioridazine-d3	374.2	129.2	78	30	ESI+
Lurasidone	493.4	166.1	40	45	ESI+
Lurasidone-d4	497.4	166.2	40	45	ESI+
Loxapine	328.2	193.1	70	44	ESI+
Loxapine-d8	336.2	193.1	70	44	ESI+
Chlorpromazine	319.1	86.1	82	27	ESI+
Chlorpromazine-d6	325.2	92.1	82	27	ESI+
Chlorprothixene	316.2	271.0	85	24	ESI+
Chlorprothixene-d6	322.2	271.1	85	24	ESI+
Ziprasidone	413.2	194.1	30	33	ESI+
Ziprasidone-d8	421.2	194.0	30	33	ESI+
Sulpiride	342.2	112.1	90	40	ESI+
Sulpiride-d3	345.2	112.1	90	40	ESI+
Trazodone	372.2	176.1	80	36	ESI+
Trazodone-d6	378.3	182.1	80	36	ESI+
N-desmetyclomipramine	301.2	242.1	38	24	ESI+
N-desmetyclomipramine-d3	304.2	242.1	32	22	ESI+
Clomipramine	315.2	86.0	66	25	ESI+
Clomipramine-d3	318.3	89.0	66	25	ESI+
N-desmethylmianserin	251.2	118.0	6	32	ESI+
Mianserin-d3	268.3	208.1	44	20	ESI+
Mianserin	265.2	208.1	48	20	ESI+
Mianserin-d3	268.3	208.1	44	20	ESI+
N-desmethylmirtazapine	252.2	195.1	50	20	ESI+
N-desmethylmirtazapine-d6	258.2	197.3	16	22	ESI+
Mirtazapine	266.2	195.1	70	20	ESI+
Mirtazapine-d3	269.2	195.1	70	20	ESI+
N-Desmethylvenlafaxine	264.3	246.2	16	10	ESI+
N-Desmethylvenlafaxine-d3	267.3	249.2	26	10	ESI+
O-Desmethylvenlafaxine	264.3	58.1	64	20	ESI+
O-Desmethylvenlafaxine-d6	270.3	64.1	64	20	ESI+
Venlafaxine	278.3	260.2	55	12	ESI+
Venlafaxine-d6	284.4	266.3	55	12	ESI+
O-Deethylreboxetine	286.2	131.1	36	18	ESI+
Reboxetine-d5	319.3	176.1	46	12	ESI+
Reboxetine	314.3	176.1	46	12	ESI+
Reboxetine-d5	319.3	176.1	46	12	ESI+
Agomelatine	244.2	185.1	60	20	ESI+
Agomelatine-d3	247.2	185.1	60	20	ESI+
Amitriptyline	278.2	105.1	40	24	ESI+
Amitriptyline-d3	281.3	105.1	12	26	ESI+
N-Desmethylamitriptyline	264.2	105.1	32	18	ESI+
N-Desmethylamitriptyline-d3	267.2	105.1	30	28	ESI+
Bupropion	240.1	184.1	26	12	ESI+
Bupropion-d9	249.2	185.1	18	12	ESI+
Hydroxybupropion	256.1	238.1	50	12	ESI+
Hydroxybupropion-d6	262.2	244.2	50	12	ESI+
Duloxetine	298.2	44.1	10	12	ESI+
Duloxetine-d7	305.2	44.2	10	12	ESI+
Doxepin	280.2	107.1	70	24	ESI+
Doxepin-d3	283.2	107.1	70	24	ESI+
N-Desmethyldoxepin	266.2	107.1	38	22	ESI+
N-Desmethyldoxepin-d3	269.2	107.1	38	25	ESI+
Vortioxetine	299.2	150.0	24	24	ESI+
Vortioxetine-13C6	305.2	156.1	36	22	ESI+
Fluvoxamine	319.2	71.1	18	14	ESI+
Fluvoxamine-d3	322.3	74.0	12	16	ESI+
Fluoxetine	310.2	148.1	24	8	ESI+
Fluoxetine-d6	316.2	154.1	10	8	ESI+
N-Desmethylfluoxetine	296.2	134.1	6	6	ESI+
N-Desmethylfluoxetine-d5	301.2	139.1	6	6	ESI+
Maprotiline	278.2	250.2	24	20	ESI+
Maprotiline-d5	283.3	255.2	36	20	ESI+
N-Desmethylmaprotiline	264.2	141.1	32	34	ESI+
Maprotiline-d5	283.3	255.2	36	20	ESI+
Moclobemide	269.2	182.0	75	28	ESI+
Moclobemide-d4	273.2	186.1	75	28	ESI+
Moclobemide N-oxindole	285.2	182.0	65	23	ESI+
Moclobemide N-oxindole-d4	289.2	186.1	65	23	ESI+
milnacipran	247.2	230.2	20	10	ESI+
milnacipran-d5	252.3	235.2	10	10	ESI+
Tianeptine	437.2	292.1	6	16	ESI+
Tianeptine-d6	443.3	292.1	14	14	ESI+
Paroxetine	330.2	192.1	46	20	ESI+
Paroxetine-d6	336.3	198.2	22	20	ESI+
Norsertraline	275.1	158.9	38	18	ESI+
Norsertraline-13C6	281.1	159.0	26	22	ESI+
Sertraline	306.2	275.1	16	12	ESI+
Sertraline-13C6	312.2	281.1	12	12	ESI+
Citalopram	325.2	109.0	70	26	ESI+
Citalopram-d6	331.3	109.0	70	24	ESI+
N-Desmethylcitalopram	311.2	262.1	24	16	ESI+
N-Desmethylcitalopram-d3	314.3	262.1	18	18	ESI+

### 2.3 Preparation of stock and working solutions

#### 2.3.1 Standard stock solution

The purity of each drug compound was different, and according to the purity stated on the certificate, the drug samples were divided into two drugs with a purity >95 % and drugs with a purity <95 %. When the purity of the drug was >95 % of the standard, 1 mg of the standard (default purity 100 %) was accurately weighed and dissolved in 1 mL of methanol. When the purity of the drug was <95 % of the standard, according to the actual purity of the quality of the conversion, and then dissolved in 1 mL of methanol.

#### 2.3.2 Configuration of standard curves and QC

##### 2.3.2.1 Standard curves

Sulpiride, chlorpromazine, agomelatine, chlorprothixene, desmethyl sertraline, sertraline, fluphenazine, haloperidol, reduced haloperidol, and flupenthixol were used in mixed concentration group 1. Blank serum was spiked with the appropriate amount of each drug to form high concentration mix 1, and the remaining five mixes were prepared from high concentration mix 1 and blank serum. The following ratios of high concentration mix 1 to blank serum were followed: 1:2, 1:5, 1:11, 1:24, and 1:99. For the rest of the drugs as mixed concentration group 2, the appropriate amount of different drugs and blank serum were mixed to prepare high concentration mixing standard 2. The remaining five mixing ratios of the high concentration mixing standard 2 to blank serum were as follows: 3:2, 1:1, 2:3, 3:7, and 1:29. Mixing standard 1 and mixing standard 2 were mixed in the ratio of 1:9, and finally a standard curve of STD1–STD6 was prepared.

##### 2.3.2.2 QC

QC preparations were obtained by diluting them from a new stock solution. QC samples comprise three concentrations covering the lowest detection limit, the range within the recommended reference concentration for the drug, and the range above the recommended concentration. The method of preparation of the three QC samples (low, medium, and high concentrations) was the same as that of standards 1, 3, and 5.

#### 2.3.3 Preparation of the internal standard precipitation solution

The appropriate amount of each drug's internal standard was precisely weighed and placed in a 1 ml volumetric flask and dissolved in methanol. The volume was made up to obtain a concentration of 1 mg/ml of internal standard reserve solution. The appropriate volume of each drug's internal standard reserve solution was then taken in a reagent bottle, and the mixed internal standard solution was prepared by mixing with methanol, and then diluted 100 times with methanol-acetonitrile reagent mixture (4:6) to obtain the internal standard precipitation solution.

### 2.4 Sample preparation

Serum sample (50 μL), QC sample(50 μL), and standard sample(50 μL) were taken in a centrifuge tube, to which was added 300 μL of internal standard precipitating solution and vortex mixed. The tube was centrifuged at a high speed at 16000*g* for 10 min at 4 °C. The supernatant (100 μL) was collected in a collection plate, to which was added 100 μL of 5 % methanol in water and mixed well. The mix was analyzed using the Waters Acquity UPLC I-Class XEVO TQS.

### 2.5 Method validation

Method validation was performed according to CLSI C62-A [22] guidelines and 9012 Guidelines for Validation of Methods for Quantitative Analysis of Biological Samples [23].

#### 2.5.1 Selectivity and specificity

Separate samples of blank human serum and serum samples with the drug-mixed standard solution added were tested. The presence of interference peaks and any effect of endogenous substances were studied.

#### 2.5.2 Linearity and sensitivity

The standard curve was plotted by linear regression with the mass concentration of the drug of interest in serum (X) as the horizontal coordinate and the peak area ratio of the drug of interest to the internal standard (Y) as the vertical coordinate. The lower limit of quantitation (LLOQ) was defined as the lowest concentration on the standard curve satisfying the following conditions S/N ≥ 10, and it was noted that the RSD of precision of 5 replicate standard curve samples was within 20 %, and the concentration deviation was within ±15 % of the theoretical value.

#### 2.5.3 Intra- and inter-day validation and accuracy

High, medium, and low concentrations of QC samples were prepared by adding standards to blank human serum. One batch was made each day for three days to investigate the intra-batch and inter-batch precision and accuracy, respectively.

#### 2.5.4 Matrix effect

For validating the matrix effect, the post sample treatment addition method was adopted. The matrix sample was taken, pre-treated, and then the target analyte was added at a certain concentration. Thus, three samples of low, medium, and high concentrations were prepared, and the measurement was repeated thrice for each concentration. The sample and the same concentration of pure solvent as the matrix sample were analyzed, and then their respective response values and the ratio of the internal standard were compared. The difference between the ratio of the matrix sample response value to the internal standard and the ratio of the solvent sample response value to the internal standard to the solvent sample response value was considered the relative matrix effect.

#### 2.5.5 Evaluation of stability

Standards were added to the blank serum to obtain specimens for stability studies at low, medium, and high concentrations. The control group was stored at −80 °C, and the other groups were placed at room temperature. They were kept at room temperature for 1, 2, 3, 5, and 7 days and then transferred to the −80 °C refrigerator. After all samples were transferred, they were tested to compare the experimental group with the control group. The samples were considered to be stable if the deviation was no more than 15 %.

#### 2.5.6 Carryover

After the high concentration sample, a blank sample was analyzed to estimate the residual carryover effect. A carryover effect was considered to be absent if residues in the blank after the high concentration sample did not exceed 20 % of the lower limit of quantification and 5 % of the internal standard.

## 3 Results

### 3.1 Selectivity and specificity

**Fig. 1:**
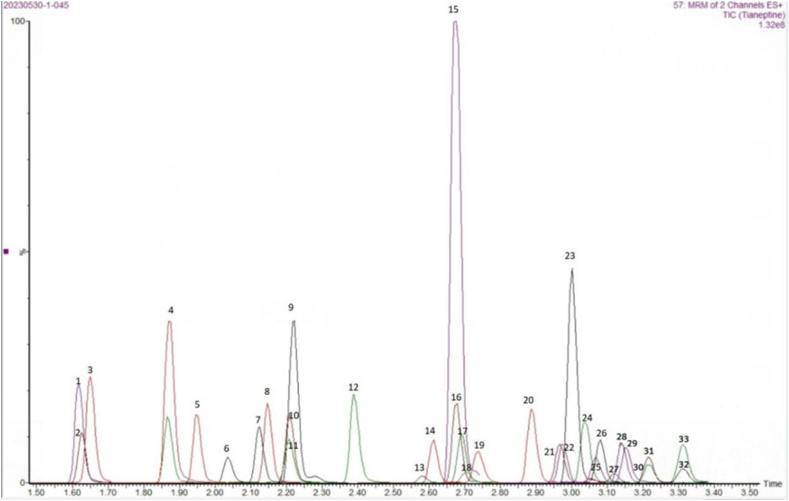
33Antidepressant drugs 1-O-desmethylvenlafaxine；2**-**moclobemide-N-Oxide；3-moclobemide；4-hydroxybupropion；5-milnacipran；6-N-desmethylvenlafaxine；7-O-desethylreboxetine；8-venlafaxine；9-bupropion；10-trazodone；11-N-desmethylmianserin；12-desmethylcitalopram；13-citalopram；14-nordoxepin；15-reboxetine；16-mianserin；17-doxepin；18-paroxetine；19-desmethylmaprotiline；20-maprotiline；21-fluvoxamine；22-agomelatine；23-duloxetine；24-norfluoxetine；25-noramitriptyline；26-tianeptine；27-amitriptyline；28-N-desmethylsertraline；29-fluoxetine；30-sertraline；31-N-desmethylclomipramine；32-clomipramine；33-vortioxetine.

**Fig. 2:**
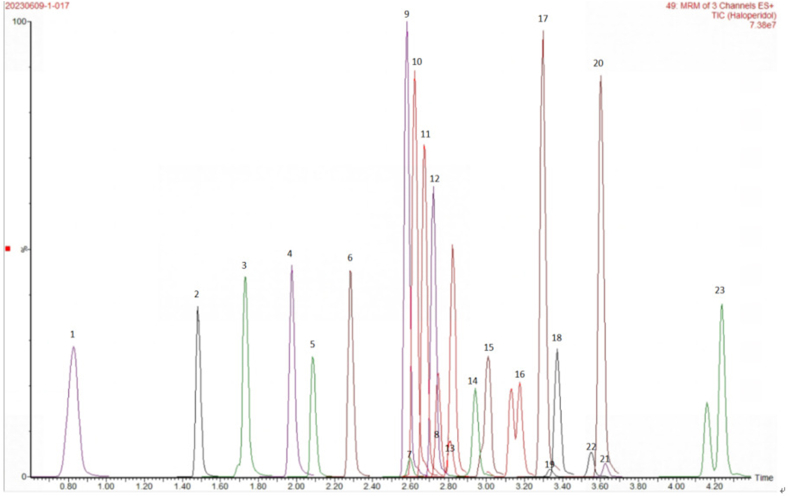
23 Antipsychotic drugs 1-sulpiride；2-amisulpride；3-N-demethylolanzapine；4-olanzapine；5-9-hydroxyrisperidone；6-risperidone；7-reduced hydrohaloperidol；8-ziprasidone；9-N-demethylclozapine；10-norquetiapine；11-clozapine；12-quetiapine；13-haloperidol；14-loxapine；15-dehydroaripiprazole；16-aripiprazole；17-chlorpromazine；18-chlorprothixene；19-perphenazine；20-thioridazine；21-flupentixol；22-fluphenazine；23-lurasidone.

**Fig. 3:**
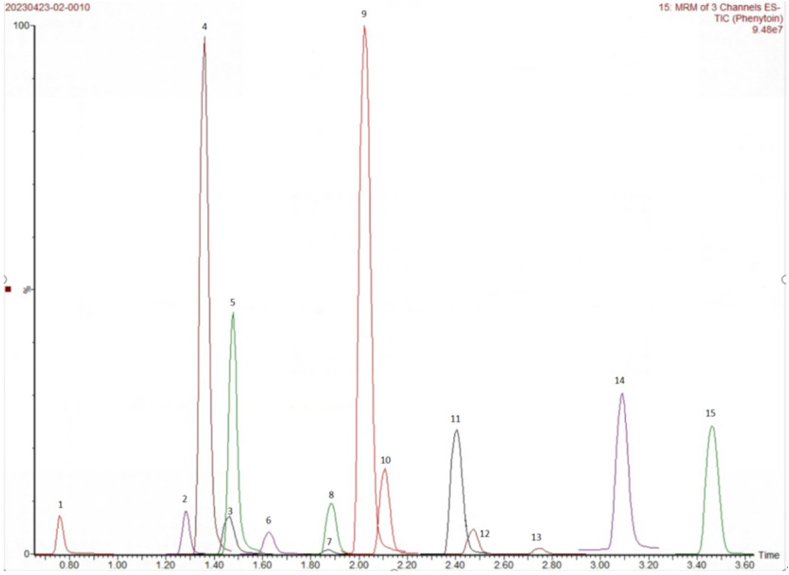
15 Anticonvulsant drugs 1-Levetiracetam；2-Primidone；3-Lamotrigine；4-Lacosamide；5-Zonisamide；6-licarbazepine；7-Phenobarbital；8-carbamazepine 10,11-epoxide；9-Oxcarbazepine；10-topiramate；11-carbamazepine；12-Phenytoin；13-Clonazepam；14-Valproic acid；15-perampanel.

The chromatographic peaks of each drug were well resolved and had a good peak shape without obvious interference from impurity peaks, and the endogenous substances in the serum did not interfere with the detection, indicating that the method is highly specific and selective (Fig. 1, Fig. 2, Fig. 3).

### 3.2 Linearity and sensitivity

The drugs analyzed exhibited good linearity in their respective concentration ranges, with correlation coefficients (r) is 0.991–0.999, concentration deviations are 0.14 %–12.61 %, and RSDs at the LLOQ levels are −10.7 %–10.9 % (Table 3).

**Table 3:** Linear range, RSD of the assay at the lower limit of quantification, and bias for 71 neuropsychotropic drugs.

Compound	Linear range （ng/ml)	Linear Correlation coefficientr	LOQ RSD （%）	Deviation from LOD （%）
Oxcarbazepine	1800∼54000	0.993	6.69	4.5
10-hydroxycarbazepine	1800∼54000	0.997	4.25	5.3
Epoxide-Carbamazepine	900∼27000	0.991	7.11	6.5
Carbamazepine	900∼27000	0.998	11.67	−7.8
Phenobarbital	1800∼54000	0.993	3.99	6.9
Primidone	900∼27000	0.994	5.77	−8.5
Phenytoin	900∼27000	0.995	6.92	5.3
Valproic acid	4500∼135000	0.994	4.84	−8.1
Lamotrigine	720∼21600	0.998	8.48	−3.4
Topiramate	900∼27000	0.992	8.66	−6.7
Levetiracetam	1800∼54000	0.995	4.56	4.5
Perampanel	45∼1350	0.996	3.15	−7.1
Zonisamide	1800∼54000	0.996	8.65	−8.2
Clonazepam	4.5–135	0.999	9.56	8.5
9-Hydroxyrisperidone	4.5–135	0.999	4.53	7.1
Risperidone	4.5–135	0.995	8.42	6.5
N-desmethylolanzapine	4.5–135	0.993	2.39	−7.2
Olanzapine	4.5–135	0.992	2.35	3.6
N-desmethylclozapine	45∼1350	0.997	2.01	5.6
Clozapine	45∼1350	0.995	2.50	−6.3
Aripiprazole	45∼1350	0.994	1.92	−10.7
Dehydroaripiprazole	45∼1350	0.999	1.22	−7.4
Amisulpride	28.8–864	0.998	3.03	10.8
Perphenazine	0.225–6.75	0.999	1.73	8.9
Fluphenazine	0.3–30	0.999	1.82	8.6
Haloperidol	0.3–30	0.999	1.32	−7.3
Reducdhaloperidol	0.3–30	0.997	1.03	5.7
Flupenthixol	0.3–30	0.993	0.46	4.5
Quetiapine	45∼1350	0.998	1.36	−8.2
Thioridazine	18∼540	0.999	1.12	4.7
Lurasidone	4.5–135	0.999	0.92	5.9
Loxapine	0.9–27	0.999	3.04	7.7
Chlorpromazine	9∼900	0.995	0.14	8.1
Chlorprothixene	6∼600	0.997	0.94	10.9
Ziprasidone	18∼540	0.998	1.37	−8.4
Sulpiride	12∼1200	0.999	1.30	−9.3
Trazodone	45∼1350	0.993	1.71	1.7
N-desmetyclomipramine	18∼540	0.998	1.32	−4.4
Clomipramine	18∼540	0.999	0.42	−5.6
N-desmethylmianserin	4.5–135	0.999	1.49	8.6
Mianserin	4.5–135	0.991	0.56	−5.3
N-desmethylmirtazapine	4.5–135	0.999	1.65	1.3
Mirtazapine	4.5–135	0.999	0.48	−2.7
N-Desmethylvenlafaxine	28.8–864	0.992	0.83	−2.4
O-Desmethylvenlafaxine	28.8–864	0.999	0.53	6.2
Venlafaxine	28.8–864	0.993	0.73	1.3
O-Deethylreboxetine	28.8–864	0.992	3.84	3.7
Reboxetine	28.8–864	0.999	0.98	−5.2
Agomelatine	6.75–675	0.999	2.93	−2.5
Amitriptyline	13.5–405	0.998	1.66	−6.2
N-Desmethylamitriptyline	13.5–405	0.999	1.52	4.9
Bupropion	90∼2700	0.999	1.02	−1.6
Hydroxybupropion	90∼2700	0.999	1.49	−3.7
Duloxetine	9∼270	0.992	4.00	−1.7
Doxepin	13.5–405	0.994	4.20	−3.7
N-Desmethyldoxepin	13.5–405	0.992	3.04	−6.9
Vortioxetine	3.6–108	0.999	3.95	2
Fluvoxamine	18∼540	0.993	3.58	5.9
Fluoxetine	45∼1350	0.993	0.64	2.6
N-Desmethylfluoxetine	45∼1350	0.999	1.09	2.4
Maprotiline	9∼270	0.992	2.64	6.5
N-Desmethylmaprotiline	9∼270	0.994	3.07	−6.6
Moclobemide	90∼2700	0.999	12.61	3.9
Moclobemide N-oxindole	90∼2700	0.999	4.82	−3.2
milnacipran	13.5–405	0.992	4.71	6.8
Tianeptine	4.5–135	0.999	6.04	5.4
Paroxetine	4.5–135	0.999	3.42	2.5
Norsertraline	4.5–450	0.999	2.11	3.1
Sertraline	4.5–450	0.999	1.89	4.2
Citalopram	9∼270	0.993	1.22	3.5
N-Desmethylcitalopram	9∼270	0.993	1.22	3.5

### 3.3 Intra and inter-day validation and accuracy

For each of the drugs analyzed, the intra-batch RSDs for the three concentration levels, low, medium, and high, were 1.2 %–9.2%and inter-batch RSDs for the three concentration levels were 1.1 %–10.6 %, and the accuracy ranged from 89.59 % to 111.18 % (Table 4).

**Table 4:** Precision and accuracy of methods for the determination of 71 neuropsychotropic drugs.

compound	concentration（ng/mL）	Intra-batch precision（n = 6）	Inter-batch precision （n = 18）	Accuracy （n = 6） %
		RSD（%）	RSD（%）	
Oxcarbazepine	1800	5.4	7.5	101.28
	21600	3.3	1.3	98.05
	32400	6.1	3.8	98.58
10-hydroxycarbazepine	1800	5.3	2.6	102.16
	21600	3.6	2.1	100.66
	32400	4.6	1.3	99.33
Epoxide-Carbamazepine	900	2	2.9	100.07
	10800	2.4	5	100.02
	16200	3.6	1.3	99.85
Carbamazepine	900	2.9	3.7	96.02
	10800	2	3.4	97.53
	16200	6.1	1.5	94.95
Phenobarbital	1800	5	3.3	95.98
	21600	5.2	2.7	96.48
	32400	5.4	1.3	96.17
Primidone	900	2.9	3.3	95.97
	10800	2.8	3.2	96.34
	16200	2.6	3.4	98.11
Phenytoin	900	7.8	3.1	102.64
	10800	4.2	4.3	97.96
	16200	5.4	5.7	97.83
Valproic acid	4500	3.4	5.6	92.30
	54000	4.3	4.4	91.90
	81000	3.2	4.8	94.30
Lamotrigine	720	4.1	4	101.27
	8640	2.7	2.2	99.52
	12960	2.1	2.9	100.44
Topiramate	900	3.5	2.1	100.67
	10800	2.1	2.1	100.71
	16200	2.7	2.6	98.98
Levetiracetam	1800	4.9	3.2	100.54
	21600	3.6	1.2	97.84
	32400	3.7	1.9	98.07
Perampanel	45	3.6	5.6	100.17
	540	5.1	7.6	98.58
	810	3.2	8.9	99.51
Zonisamide	1800	4.2	3.5	101.89
	21600	5.5	2.1	100.20
	32400	9.2	3.4	99.36
Clonazepam	4.5	6.9	3.5	100.71
	54	6	2.1	100.37
	81	6.5	4.3	99.22
9-Hydroxyrisperidone	4.5	2.5	2.8	99.78
	54	2.4	4.3	99.23
	81	5.3	1.7	97.84
Risperidone	4.5	4.8	5.7	99.65
	54	2.8	3.4	98.33
	81	2.6	3.5	97.54
N-desmethylolanzapine	4.5	2.6	2.9	99.93
	54	3.8	5.4	98.36
	81	3.5	3.1	99.70
Olanzapine	4.5	4	7.1	102.15
	54	2.7	3.1	100.74
	81	3	3.6	98.59
N-desmethylclozapine	45	6.2	6.5	99.06
	540	2.1	3.2	98.70
	810	2.4	6.2	98.21
Clozapine	45	1.5	8	101.21
	540	2.2	8.1	100.30
	810	3.3	9.1	99.09
Aripiprazole	45	1.5	1.3	100.06
	540	2.3	3.8	98.57
	810	1.5	2.8	97.86
Dehydroaripiprazole	45	2.6	1.9	103.08
	540	1.6	2.2	99.81
	810	1.3	1.8	95.37
Amisulpride	28.8	1.4	2.6	99.59
	345.6	1.5	1.4	102.63
	518.4	2.2	3.1	96.66
Perphenazine	0.225	3.7	2	103.85
	2.7	3.2	2.4	101.53
	4.05	3.9	2.2	97.50
Fluphenazine	0.3	3.3	2.8	98.62
	2.5	3.1	3.6	95.08
	10	2.4	2.2	95.62
Haloperidol	0.3	2.6	4.3	100.17
	2.5	3.6	5.4	96.55
	10	5.2	2.2	97.61
Reducdhaloperidol	0.3	2.7	1.5	100.00
	2.5	2.9	1.1	97.29
	10	3.7	1.4	100.91
Flupenthixol	0.3	2.7	6.3	102.84
	2.5	4.1	9.2	99.41
	10	4.2	8.2	99.01
Quetiapine	45	4.6	9.3	102.44
	540	3.2	9.5	99.50
	810	4.1	8.6	99.07
Thioridazine	18	2.5	3.6	101.80
	216	4.6	9.8	96.75
	324	3.2	5.9	96.70
Lurasidone	4.5	8.3	7.2	100.60
	54	5.7	7.7	98.76
	81	4.9	8.8	97.75
Loxapine	0.9	2.8	7.7	103.45
	10.8	5.5	8.9	99.95
	16.2	5.4	9.4	99.80
Chlorpromazine	9	4.6	8.9	99.82
	75	4.2	5.3	100.69
	300	3.8	9.3	99.24
Chlorprothixene	6	2.4	8.3	99.32
	50	4.6	10.6	102.11
	200	5.2	10.6	99.30
Ziprasidone	18	4.1	10.4	98.83
	216	2.6	10.1	99.94
	324	2.9	9.6	98.09
Sulpiride	12	2.6	10.2	102.83
	100	6.4	9.3	97.56
	400	7.3	6.5	99.74
Trazodone	45	6.7	7.8	100.40
	540	3.8	9.3	100.21
	810	4.9	10.1	100.26
N-desmetyclomipramine	18	3.3	7.2	98.93
	216	2.4	7.3	99.09
	324	2.6	7.5	101.04
Clomipramine	18	2.3	2.8	101.46
	216	3	3.7	99.72
	324	4.1	2.4	99.26
N-desmethylmianserin	4.5	3.1	3.8	100.88
	54	2.6	7.3	100.28
	81	1.5	2.6	99.32
Mianserin	4.5	7.1	2.8	100.21
	54	4.9	7.2	99.11
	81	3.4	5.2	96.38
N-desmethylmirtazapine	4.5	3.1	7.8	99.93
	54	2.5	5.9	98.60
	81	3.9	4.2	101.03
Mirtazapine	4.5	3.8	6.1	101.33
	54	3.1	3.7	99.60
	81	1.4	5.7	97.87
N-Desmethylvenlafaxine	28.8	2.1	1.6	101.34
	345.6	3.9	5.3	98.71
	518.4	4.8	8.2	100.71
O-Desmethylvenlafaxine	28.8	3.5	4.6	101.08
	345.6	1.2	5.7	100.10
	518.4	1.5	4.4	99.28
Venlafaxine	28.8	3.1	5.2	93.82
	345.6	5.6	7.5	91.87
	518.4	6.4	3.8	93.53
O-Deethylreboxetine	28.8	5.4	5.2	93.42
	345.6	2.2	5.7	92.63
	518.4	5.6	7.1	93.48
Reboxetine	28.8	4.6	5.7	99.07
	345.6	1.7	2.7	99.51
	518.4	3.5	3.1	103.92
Agomelatine	6.75	4.6	5.5	105.48
	56.25	1.5	5.7	102.83
	225	3.8	5.6	99.91
Amitriptyline	13.5	5.8	5.2	98.68
	162	4.3	6.1	95.15
	243	5.8	7.8	95.46
N-Desmethylamitriptyline	13.5	4.9	7.5	99.65
	162	6.7	8.2	95.43
	243	2.5	4.1	94.32
Bupropion	90	4	5.2	100.32
	1080	3.4	4.5	94.20
	1620	5.6	4.9	94.78
Hydroxybupropion	90	3.4	7.3	95.73
	1080	4.1	7.9	99.79
	1620	3.2	3.9	101.24
Duloxetine	9	2.1	5.4	103.51
	108	3.1	4.3	99.85
	162	2.6	6.9	100.41
Doxepin	13.5	3.1	7.4	103.59
	162	3.2	5.7	99.92
	243	4.4	9.5	103.67
N-Desmethyldoxepin	13.5	5.5	8.3	98.61
	162	6.1	6.7	107.49
	243	1.7	5.5	94.51
Vortioxetine	3.6	2.7	4.3	97.93
	43.2	5.2	8.3	108.85
	64.8	3.2	7.3	104.77
Fluvoxamine	18	4.5	6.8	94.72
	216	4.3	5.1	95.67
	324	7.3	4.4	97.48
Fluoxetine	45	4.3	5.6	102.20
	540	4.7	6.3	100.76
	810	3.1	7.5	103.17
N-Desmethylfluoxetine	45	4.1	7.5	104.70
	540	3.1	5.5	101.56
	810	3.4	4.2	101.56
Maprotiline	9	3.2	1.1	100.00
	108	3.2	1.3	95.24
	162	3.1	4.9	105.56
N-Desmethylmaprotiline	9	4.1	3.1	96.97
	108	7.4	5.5	101.85
	162	2.2	5.6	103.92
Moclobemide	90	4	3.2	105.56
	1080	8.5	2.1	98.43
	1620	3.6	2.5	95.00
Moclobemide N-oxindole	90	3.8	1.4	96.92
	1080	3.9	6.4	94.60
	1620	3.6	1.4	100.50
milnacipran	13.5	6	1.8	97.69
	162	8.7	6.7	100.64
	243	7	3.1	98.50
Tianeptine	4.5	7	6.9	94.96
	54	8.5	3.4	111.18
	81	7.1	1.9	109.32
Paroxetine	4.5	7.9	2.4	108.37
	54	7.2	1.9	102.04
	81	7.2	4.6	96.51
Norsertraline	4.5	3.3	1.2	95.49
	37.5	3.6	3.2	92.55
	150	5.7	6.4	89.59
Sertraline	4.5	3.1	4.3	109.40
	37.5	2.5	3.6	98.87
	150	1.5	3.5	98.05
Citalopram	9	5.2	3.9	101.12
	108	4.8	2.1	99.81
	162	6.5	4.3	100.03
N-Desmethylcitalopram	9	8	7.4	101.07
	108	5.7	6.6	99.23
	162	6.1	5.7	103.24

### 3.4 Matrix effect

The relative matrix effect results for each analyte were −14.98 %–19.67 % of the required limit (Table 5).

**Table 5:** Matrix effect evaluations of 71 neuropsychotropic drugs.

compound	Mean value of detection（ug/mL）	CV	relative matrix effect
Oxcarbazepine	Mean value of detection（ug/mL）	CV	relative matrix effect
L	6.210	1.40 %	
M	20.029	0.37 %	
H	31.268	0.70 %	
Serum-L	6.356	0.33 %	−2.84 %
Serum-M	19.698	1.08 %	−2.67 %
Serum-H	31.824	0.59 %	−2.31 %
10-hydroxycarbazepine	Mean value of detection （ug/mL）	CV	relative matrix effect
L	6.221	1.29 %	
M	20.156	0.39 %	
H	30.992	1.09 %	
Serum-L	6.461	2.34 %	−0.20 %
Serum-M	19.881	0.53 %	−2.41 %
Serum-H	32.332	0.49 %	−3.26 %
Epoxide-Carbamazepine	Mean value of detection（ug/mL）	CV	relative matrix effect
L	3.133	4.18 %	
M	10.038	1.24 %	
H	15.628	0.53 %	
Serum-L	3.247	3.51 %	3.50 %
Serum-M	9.724	3.32 %	−3.23 %
Serum-H	16.032	1.07 %	4.86 %
Carbamazepine	Mean value of detection（ug/mL）	CV	relative matrix effect
L	3.129	0.94 %	
M	10.165	0.74 %	
H	15.669	1.93 %	
Serum-L	3.163	0.84 %	−1.74 %
Serum-M	9.941	0.86 %	4.88 %
Serum-H	15.943	0.74 %	0.72 %
Phenobarbital	Mean value of detection（ug/mL）	CV	relative matrix effect
L	7.514	1.19 %	
M	24.365	1.18 %	
H	37.779	0.52 %	
Serum-L	7.490	0.74 %	−0.32 %
Serum-M	23.827	1.02 %	−2.26 %
Serum-H	38.066	0.36 %	4.86 %
Primidone	Mean value of detection（ug/mL）	CV	relative matrix effect
L	3.890	0.96 %	
M	12.198	1.59 %	
H	18.271	1.64 %	
Serum-L	3.963	1.93 %	1.84 %
Serum-M	12.238	0.88 %	0.32 %
Serum-H	19.001	0.46 %	4.86 %
Phenytoin	Mean value of detection（ug/mL）	CV	relative matrix effect
L	3.781	1.06 %	
M	12.243	0.47 %	
H	18.760	0.36 %	
Serum-L	3.820	0.71 %	1.01 %
Serum-M	11.956	0.53 %	−2.40 %
Serum-H	19.144	0.26 %	4.86 %
Valproic acid	Mean value of detection（ug/mL）	CV	relative matrix effect
L	18.718	0.18 %	
M	61.147	0.18 %	
H	93.751	0.32 %	
Serum-L	19.159	0.39 %	2.31 %
Serum-M	59.906	0.54 %	−2.07 %
Serum-H	95.810	0.12 %	4.86 %
Lamotrigine	Mean value of detection（ug/mL）	CV	relative matrix effect
L	3.051	1.00 %	
M	10.049	1.31 %	
H	15.854	2.38 %	
Serum-L	3.183	2.52 %	4.14 %
Serum-M	10.186	3.34 %	1.35 %
Serum-H	16.020	1.43 %	4.86 %
Topiramate	Mean value of detection（ug/mL）	CV	relative matrix effect
L	2.488	1.09 %	
M	8.160	2.48 %	
H	12.727	2.07 %	
Serum-L	2.576	2.07 %	3.43 %
Serum-M	8.021	0.96 %	−1.73 %
Serum-H	12.727	2.44 %	4.86 %
Levetiracetam	Mean value of detection（ug/mL）	CV	relative matrix effect
L	16.092	0.57 %	
M	48.625	0.90 %	
H	72.223	0.57 %	
Serum-L	16.881	0.81 %	4.68 %
Serum-M	49.789	1.16 %	2.34 %
Serum-H	77.417	0.87 %	4.86 %
Perampanel	Mean value of detection（ug/mL）	CV	relative matrix effect
L	3.108	0.42 %	
M	10.129	0.55 %	
H	15.633	0.43 %	
Serum-L	3.160	0.13 %	1.64 %
Serum-M	9.923	0.41 %	−2.08 %
Serum-H	15.809	0.43 %	4.86 %
Zonisamide	Mean value of detection（ug/mL）	CV	relative matrix effect
L	0.013	1.96 %	
M	0.041	0.50 %	
H	0.063	1.36 %	
Serum-L	0.013	1.93 %	1.53 %
Serum-M	0.041	1.34 %	−0.08 %
Serum-H	0.064	1.03 %	4.86 %
Clonazepam	Mean value of detection（ug/mL）	CV	relative matrix effect
L	0.159	0.68 %	
M	0.514	0.64 %	
H	0.787	0.42 %	
Serum-L	0.164	0.76 %	3.09 %
Serum-M	0.507	0.21 %	−1.40 %
Serum-H	0.804	0.46 %	4.86 %
9-Hydroxyrisperidone	Mean value of detection（ng/mL）	CV	relative matrix effect
L	4.24	0.72 %	
M	36.32	1.05 %	
H	115.27	0.08 %	
Serum-L	4.21	0.65 %	−0.56 %
Serum-M	36.86	0.98 %	1.50 %
Serum-H	116.45	0.54 %	1.02 %
Risperidone	Mean value of detection（ng/mL）	CV	relative matrix effect
L	4.35	0.32 %	
M	36.10	0.84 %	
H	115.91	0.35 %	
Serum-L	4.34	2.33 %	−0.35 %
Serum-M	37.12	0.85 %	2.82 %
Serum-H	118.06	0.36 %	1.85 %
N-desmethylolanzapine	Mean value of detection（ng/mL）	CV	relative matrix effect
L	5.55	1.36 %	
M	45.98	0.21 %	
H	147.77	0.38 %	
Serum-L	5.63	0.56 %	1.45 %
Serum-M	50.26	0.31 %	9.30 %
Serum-H	154.58	0.53 %	4.61 %
Olanzapine	Mean value of detection（ng/mL）	CV	relative matrix effect
L	5.50	0.62 %	
M	45.99	0.27 %	
H	147.50	1.05 %	
Serum-L	5.27	0.36 %	−4.04 %
Serum-M	47.56	0.76 %	3.42 %
Serum-H	151.52	1.02 %	2.73 %
N-demethylclozapine	Mean value of detection（ng/mL）	CV	relative matrix effect
L	37.49	0.66 %	
M	308.45	2.24 %	
H	965.00	1.18 %	
Serum-L	37.37	2.23 %	−0.32 %
Serum-M	340.64	0.79 %	10.43 %
Serum-H	1059.36	1.08 %	9.78 %
Clozapine	Mean value of detection（ng/mL）	CV	relative matrix effect
L	36.45	0.29 %	
M	301.74	0.49 %	
H	965.75	0.95 %	
Serum-L	36.60	0.13 %	0.43 %
Serum-M	323.11	1.07 %	7.08 %
Serum-H	983.02	0.86 %	1.79 %
Aripiprazole	Mean value of detection（ng/mL）	CV	relative matrix effect
L	36.85	4.44 %	
M	330.32	0.91 %	
H	946.64	1.29 %	
Serum-L	38.58	2.01 %	4.70 %
Serum-M	367.49	2.25 %	11.25 %
Serum-H	1102.79	1.17 %	16.50 %
Dehydro aripiprazole	Mean value of detection（ng/mL）	CV	relative matrix effect
L	36.38	0.57 %	
M	314.68	1.79 %	
H	949.83	2.12 %	
Serum-L	37.19	2.15 %	2.24 %
Serum-M	338.95	1.72 %	7.71 %
Serum-H	1009.95	0.34 %	6.33 %
Amisulpride	Mean value of detection（ng/mL）	CV	relative matrix effect
L	22.67	4.79 %	
M	196.92	0.88 %	
H	615.74	5.00 %	
Serum-L	22.03	3.07 %	−2.84 %
Serum-M	191.66	2.97 %	−2.67 %
Serum-H	601.53	3.50 %	−2.31 %
Perphenazine	Mean value of detection（ng/mL）	CV	relative matrix effect
L	0.16	3.55 %	
M	1.63	1.24 %	
H	4.85	2.56 %	
Serum-L	0.16	2.46 %	−4.45 %
Serum-M	1.68	1.44 %	2.96 %
Serum-H	4.85	2.95 %	−0.01 %
Fluphenazine	Mean value of detection（ng/mL）	CV	relative matrix effect
L	23.32	1.83 %	
M	193.16	1.24 %	
H	621.68	1.08 %	
Serum-L	22.95	2.08 %	−1.58 %
Serum-M	199.94	1.08 %	3.51 %
Serum-H	642.02	1.91 %	3.27 %
Haloperidol	Mean value of detection（ng/mL）	CV	relative matrix effect
STD9	18.08	2.29 %	
L	0.52	3.68 %	
M	4.51	2.03 %	
H	14.67	0.10 %	
Serum-L	0.51	2.22 %	−1.74 %
Serum-M	4.73	0.57 %	4.88 %
Serum-H	14.78	1.02 %	0.72 %
Reduced haloperidol	Mean value of detection（ng/mL）	CV	relative matrix effect
L	0.57	3.81 %	
M	4.62	0.37 %	
H	14.38	1.18 %	
Serum-L	0.57	4.15 %	0.05 %
Serum-M	4.90	3.85 %	6.07 %
Serum-H	14.76	3.16 %	2.61 %
Flupentixol	Mean value of detection（ng/mL）	CV	relative matrix effect
L	0.55	1.86 %	
M	4.69	0.53 %	
H	14.52	2.04 %	
Serum-L	0.56	1.06 %	0.37 %
Serum-M	5.01	3.92 %	6.87 %
Serum-H	16.33	0.48 %	12.50 %
Quetiapine	Mean value of detection（ng/mL）	CV	relative matrix effect
L	36.96	0.40 %	
M	297.07	0.85 %	
H	964.95	0.77 %	
Serum-L	36.79	1.74 %	−0.44 %
Serum-M	323.70	0.43 %	8.96 %
Serum-H	1023.30	0.24 %	6.05 %
Thioridazine	Mean value of detection（ng/mL）	CV	relative matrix effect
L	15.19	0.78 %	
M	123.66	0.35 %	
H	402.87	1.59 %	
Serum-L	15.63	0.98 %	2.95 %
Serum-M	136.49	1.70 %	10.38 %
Serum-H	418.93	0.27 %	3.99 %
Lurasidone	Mean value of detection（ng/mL）	CV	relative matrix effect
L	4.39	1.86 %	
M	36.54	0.37 %	
H	115.66	0.77 %	
Serum-L	4.01	0.33 %	−8.63 %
Serum-M	33.28	0.75 %	−8.91 %
Serum-H	98.34	0.47 %	−14.98 %
Loxapine	Mean value of detection（ng/mL）	CV	relative matrix effect
L	0.77	3.27 %	
M	5.85	3.30 %	
H	19.14	2.26 %	
Serum-L	0.80	0.30 %	4.30 %
Serum-M	6.50	2.98 %	10.95 %
Serum-H	21.01	1.55 %	9.77 %
Chlorpromazine	Mean value of detection（ng/mL）	CV	relative matrix effect
L	22.72	1.59 %	
M	185.10	0.24 %	
H	575.94	2.09 %	
Serum-L	21.94	1.21 %	−3.43 %
Serum-M	195.18	1.49 %	5.45 %
Serum-H	617.85	1.87 %	7.28 %
Chlorprothixene	Mean value of detection（ng/mL）	CV	relative matrix effect
L	14.56	1.20 %	
M	120.76	1.58 %	
H	390.32	1.01 %	
Serum-L	14.87	0.87 %	2.10 %
Serum-M	134.57	1.63 %	11.44 %
Serum-H	417.58	1.06 %	6.99 %
Ziprasidone	Mean value of detection（ng/mL）	CV	relative matrix effect
L	14.43	1.20 %	
M	119.72	1.14 %	
H	383.86	1.10 %	
Serum-L	13.87	1.78 %	−3.86 %
Serum-M	121.51	0.92 %	1.49 %
Serum-H	389.29	0.89 %	1.41 %
Sulpiride	Mean value of detection（ng/mL）	CV	relative matrix effect
L	36.68	0.25 %	
M	303.97	0.15 %	
H	961.68	0.14 %	
Serum-L	35.85	0.29 %	−2.27 %
Serum-M	319.83	0.61 %	5.22 %
Serum-H	1003.51	0.21 %	4.35 %
Trazodone	Mean value of detection（ng/mL）	CV	relative matrix effect
L	42.42	1.46 %	
M	366.40	0.69 %	
H	1148.83	0.25 %	
Serum-L	40.82	0.28 %	−3.77 %
Serum-M	372.98	0.90 %	1.80 %
Serum-H	1154.11	0.47 %	0.46 %
N-desmetyclomipramine	Mean value of detection（ng/mL）	CV	relative matrix effect
L	42.71	4.23 %	
M	363.14	0.49 %	
H	1173.04	1.49 %	
Serum-L	40.50	2.78 %	−5.17 %
Serum-M	376.07	1.99 %	3.56 %
Serum-H	1200.17	1.63 %	2.31 %
Clomipramine	Mean value of detection（ng/mL）	CV	relative matrix effect
L	16.29	0.57 %	
M	136.38	1.25 %	
H	435.37	0.80 %	
Serum-L	17.75	1.68 %	9.01 %
Serum-M	157.42	1.74 %	15.43 %
Serum-H	501.19	0.73 %	15.12 %
N-desmethylmianserin	Mean value of detection（ng/mL）	CV	relative matrix effect
L	5.12	0.22 %	
M	42.75	1.33 %	
H	136.13	1.46 %	
Serum-L	5.30	2.28 %	3.48 %
Serum-M	45.83	1.66 %	7.20 %
Serum-H	140.67	0.96 %	3.33 %
Mianserin	Mean value of detection（ng/mL）	CV	relative matrix effect
L	5.25	2.34 %	
M	42.82	1.78 %	
H	134.36	1.72 %	
Serum-L	4.90	3.40 %	−6.66 %
Serum-M	44.13	2.37 %	3.05 %
Serum-H	134.34	1.34 %	−0.02 %
N-desmethylmirtazapine	Mean value of detection（ng/mL）	CV	relative matrix effect
M	49.27	0.32 %	
H	153.08	0.94 %	
Serum-L	5.06	0.75 %	−13.59 %
Serum-M	46.37	0.23 %	−5.89 %
Serum-H	146.30	0.90 %	−4.43 %
Mirtazapine	Mean value of detection（ng/mL）	CV	relative matrix effect
L	5.98	1.71 %	
M	48.73	0.43 %	
H	156.23	0.93 %	
Serum-L	6.03	4.39 %	0.86 %
Serum-M	51.32	1.05 %	5.31 %
Serum-H	162.55	0.72 %	4.05 %
N-desmethylvenlafaxine	Mean value of detection（ng/mL）	CV	relative matrix effect
L	29.96	1.70 %	
M	247.49	1.09 %	
H	776.85	1.21 %	
Serum-L	35.27	0.25 %	17.74 %
Serum-M	212.05	0.49 %	−14.32 %
Serum-H	877.97	1.45 %	13.02 %
O-desmethylvenlafaxine	Mean value of detection（ng/mL）	CV	relative matrix effect
L	29.69	0.91 %	
M	244.54	0.50 %	
H	779.08	0.36 %	
Serum-L	32.57	1.03 %	9.72 %
Serum-M	279.26	0.36 %	14.20 %
Serum-H	854.48	0.59 %	9.68 %
Venlafaxine	Mean value of detection（ng/mL）	CV	relative matrix effect
L	28.93	2.24 %	
M	231.65	0.60 %	
H	807.13	0.31 %	
Serum-L	27.45	0.26 %	−5.14 %
Serum-M	234.24	1.10 %	1.12 %
Serum-H	960.67	1.18 %	19.02 %
O-Deethylreboxetine	Mean value of detection（ng/mL）	CV	relative matrix effect
L	26.05	0.84 %	
M	203.86	0.42 %	
H	691.37	1.30 %	
Serum-L	26.04	4.11 %	−0.05 %
Serum-M	211.32	1.86 %	3.66 %
Serum-H	718.15	0.84 %	3.87 %
Reboxetine	Mean value of detection（ng/mL）	CV	relative matrix effect
L	25.67	1.45 %	
M	212.92	0.95 %	
H	674.22	1.73 %	
Serum-L	26.24	0.17 %	2.19 %
Serum-M	227.83	0.50 %	7.00 %
Serum-H	738.36	1.36 %	9.51 %
Agomelatine	Mean value of detection（ng/mL）	CV	relative matrix effect
L	21.85	3.60 %	
M	178.46	1.68 %	
H	577.66	0.49 %	
Serum-L	23.30	1.13 %	6.66 %
Serum-M	200.68	1.07 %	12.45 %
Serum-H	634.12	1.14 %	9.77 %
Amitriptyline	Mean value of detection（ng/mL）	CV	relative matrix effect
L	11.17	1.98 %	
M	91.79	1.48 %	
H	290.38	0.22 %	
Serum-L	10.70	1.11 %	−4.22 %
Serum-M	99.71	2.77 %	8.63 %
Serum-H	305.00	0.35 %	5.04 %
N-Desmethylamitriptyline	Mean value of detection（ng/mL）	CV	relative matrix effect
L	10.86	1.78 %	
M	93.01	1.35 %	
H	286.74	1.01 %	
Serum-L	10.88	3.52 %	0.16 %
Serum-M	95.61	1.60 %	2.80 %
Serum-H	291.25	1.72 %	1.57 %
Bupropion	Mean value of detection（ng/mL）	CV	relative matrix effect
L	71.64	1.35 %	
M	575.45	0.90 %	
H	2006.70	0.46 %	
Serum-L	76.67	3.21 %	7.03 %
Serum-M	625.35	0.14 %	8.67 %
Serum-H	2105.46	1.16 %	4.92 %
Hydroxybupropion	Mean value of detection（ng/mL）	CV	relative matrix effect
L	73.89	0.67 %	
M	649.46	0.99 %	
H	1933.57	0.82 %	
Serum-L	83.71	0.40 %	13.30 %
Serum-M	768.69	0.68 %	18.36 %
Serum-H	2098.47	0.69 %	8.53 %
Duloxetine	Mean value of detection（ng/mL）	CV	relative matrix effect
L	8.30	6.19 %	
M	71.71	3.67 %	
H	231.06	0.88 %	
Serum-L	8.42	5.76 %	1.42 %
Serum-M	75.38	5.46 %	5.12 %
Serum-H	235.26	2.13 %	1.82 %
Doxepin	Mean value of detection（ng/mL）	CV	relative matrix effect
L	10.97	1.32 %	
M	93.05	5.18 %	
H	290.03	0.27 %	
Serum-L	10.91	1.77 %	−0.54 %
Serum-M	96.07	0.99 %	3.25 %
Serum-H	306.58	0.65 %	5.71 %
N-Desmethyldoxepin	Mean value of detection（ng/mL）	CV	relative matrix effect
L	12.14	15.05 %	
M	91.43	0.53 %	
H	291.20	0.26 %	
Serum-L	10.75	1.41 %	−11.46 %
Serum-M	95.74	0.66 %	4.72 %
Serum-H	301.25	1.17 %	3.45 %
Vortioxetine	Mean value of detection（ng/mL）	CV	relative matrix effect
L	2.89	0.75 %	
M	24.10	1.50 %	
H	77.83	1.47 %	
Serum-L	2.85	2.57 %	−1.45 %
Serum-M	25.32	2.07 %	5.07 %
Serum-H	81.03	0.77 %	4.11 %
Fluvoxamine	Mean value of detection（ng/mL）	CV	relative matrix effect
L	17.78	0.64 %	
M	149.26	1.74 %	
H	483.93	0.43 %	
Serum-L	16.90	0.78 %	−4.95 %
Serum-M	153.93	0.80 %	3.13 %
Serum-H	479.97	1.17 %	−0.82 %
Fluoxetine	Mean value of detection（ng/mL）	CV	relative matrix effect
L	37.56	1.80 %	
M	312.08	3.00 %	
H	992.46	1.86 %	
Serum-L	37.12	2.48 %	−1.18 %
Serum-M	338.94	2.67 %	8.61 %
Serum-H	1013.17	1.19 %	2.09 %
N-Desmethylfluoxetine	Mean value of detection（ng/mL）	CV	relative matrix effect
L	36.80	3.47 %	
M	305.70	2.86 %	
H	941.12	1.45 %	
Serum-L	38.32	1.17 %	4.13 %
Serum-M	341.40	2.46 %	11.68 %
Serum-H	1066.49	1.12 %	13.32 %
Maprotiline	Mean value of detection（ng/mL）	CV	relative matrix effect
L	8.05	1.41 %	
M	66.42	1.09 %	
H	211.71	3.02 %	
Serum-L	8.13	1.64 %	1.01 %
Serum-M	70.53	0.30 %	6.19 %
Serum-H	223.60	2.18 %	5.61 %
N-Desmethylmaprotiline	Mean value of detection（ng/mL）	CV	relative matrix effect
L	8.54	3.60 %	
M	63.16	2.92 %	
H	211.61	1.81 %	
Serum-L	9.30	2.75 %	8.92 %
Serum-M	70.28	1.08 %	11.28 %
Serum-H	250.94	2.61 %	18.59 %
Moclobemide	Mean value of detection（ng/mL）	CV	relative matrix effect
L	72.67	0.61 %	
M	617.12	0.99 %	
H	1926.88	0.97 %	
Serum-L	72.91	1.07 %	0.33 %
Serum-M	639.48	0.39 %	3.62 %
Serum-H	1985.84	0.82 %	3.06 %
Moclobemide-N-Oxide	Mean value of detection（ng/mL）	CV	relative matrix effect
L	73.44	0.98 %	
M	628.50	0.44 %	
H	1913.76	0.37 %	
Serum-L	76.54	1.99 %	4.23 %
Serum-M	698.42	0.85 %	11.12 %
Serum-H	2103.05	0.34 %	9.89 %
milnacipran	Mean value of detection（ng/mL）	CV	relative matrix effect
L	45.33	3.32 %	
M	366.44	0.96 %	
H	1100.77	0.79 %	
Serum-L	51.59	2.06 %	13.81 %
Serum-M	435.71	0.83 %	18.91 %
Serum-H	1251.56	0.67 %	13.70 %
Tianeptine	Mean value of detection（ng/mL）	CV	relative matrix effect
L	5.78	1.20 %	
M	48.01	2.45 %	
H	152.46	1.36 %	
Serum-L	5.71	0.67 %	−1.28 %
Serum-M	50.08	0.53 %	4.32 %
Serum-H	157.13	3.04 %	3.07 %
Paroxetine	Mean value of detection（ng/mL）	CV	relative matrix effect
L	4.25	4.00 %	
M	35.99	1.60 %	
H	117.02	0.56 %	
Serum-L	4.70	2.03 %	10.57 %
Serum-M	43.07	2.48 %	19.67 %
Serum-H	133.72	1.94 %	14.27 %
Norsertraline	Mean value of detection（ng/mL）	CV	relative matrix effect
L	41.68	3.63 %	
M	363.16	4.53 %	
H	1161.35	5.07 %	
Serum-L	44.27	3.30 %	6.21 %
Serum-M	388.77	3.20 %	7.05 %
Serum-H	1213.23	0.98 %	4.47 %
Sertraline	Mean value of detection（ng/mL）	CV	relative matrix effect
L	10.64	3.00 %	
M	87.30	1.90 %	
H	288.47	1.22 %	
Serum-L	12.05	5.66 %	13.31 %
Serum-M	102.49	2.39 %	17.41 %
Serum-H	341.99	2.18 %	18.55 %
Citalopram	Mean value of detection（ng/mL）	CV	relative matrix effect
L	7.92	0.82 %	
M	67.10	1.79 %	
H	212.04	0.30 %	
Serum-L	8.10	1.53 %	2.38 %
Serum-M	72.19	0.94 %	7.58 %
Serum-H	226.08	0.64 %	6.62 %
N-Desmethylcitalopram	Mean value of detection（ng/mL）	CV	relative matrix effect
L	5.74	0.51 %	
M	48.23	0.06 %	
H	153.41	0.18 %	
Serum-L	5.47	2.38 %	−4.64 %
Serum-M	42.32	0.99 %	−12.25 %
Serum-H	134.13	0.84 %	−12.57 %

### 3.5 Stability evaluation

All compounds except moclobemide N-oxindole left at room temperature for 7 days were −13.25 %–14.91 %15 % deviation from the control (Table 6).

**Table 6:** Stability evaluations of 71 neuropsychotropic drugs.

Compound	Mean value of detection（ug/mL）	CV	Deviation from control group
Oxcarbazepine			
L-CONTROL GROUP	6.215	2.51 %	
L-1d	6.245	2.64 %	0.48 %
L-2d	6.315	2.58 %	1.61 %
L-3d	6.542	2.69 %	5.26 %
L-5d	6.584	2.57 %	5.94 %
L-7d	6.235	2.68 %	0.32 %
M-CONTROL GROUP	20.154	2.61 %	
M-1d	20.165	2.84 %	0.05 %
M-2d	20.487	2.91 %	1.65 %
M-3d	20.698	2.06 %	2.70 %
M-5d	20.651	1.87 %	2.47 %
M-7d	20.512	1.95 %	1.78 %
H-CONTROL GROUP	31.548	1.54 %	
H-1d	31.573	1.64 %	0.08 %
H-2d	31.654	1.65 %	0.34 %
H-3d	31.587	1.62 %	0.12 %
H-5d	31.256	1.54 %	−0.93 %
H-7d	31.265	1.02 %	−0.90 %
10-hydroxycarbazepine	Mean value of detection（ug/mL）	CV	
L-CONTROL GROUP	6.345	2.33 %	
L-1d	6.445	2.34 %	1.58 %
L-2d	6.215	2.78 %	−2.05 %
L-3d	6.342	2.61 %	−0.05 %
L-5d	6.234	2.44 %	−1.75 %
L-7d	6.202	2.35 %	−2.25 %
M-CONTROL GROUP	20.261	2.41 %	
M-1d	20.115	2.64 %	−0.72 %
M-2d	20.367	2.71 %	0.52 %
M-3d	20.088	2.86 %	−0.85 %
M-5d	20.161	1.94 %	−0.49 %
M-7d	20.472	2.51 %	1.04 %
H-CONTROL GROUP	32.112	1.77 %	
H-1d	31.654	1.86 %	−1.43 %
H-2d	31.884	1.69 %	−0.71 %
H-3d	31.467	2.04 %	−2.01 %
H-5d	32.346	2.11 %	0.73 %
H-7d	32.335	1.95 %	0.69 %
Epoxide-Carbamazepine	Mean value of detection（ug/mL）	CV	
L-CONTROL GROUP	3.098	2.61 %	
L-1d	3.057	3.51 %	−1.32 %
L-2d	3.058	3.45 %	−1.29 %
L-3d	3.059	2.98 %	−1.26 %
L-5d	3.065	2.65 %	−1.07 %
L-7d	3.014	2.61 %	−2.71 %
M-CONTROL GROUP	10.287	2.51 %	
M-1d	10.687	2.61 %	3.89 %
M-2d	10.548	2.05 %	2.54 %
M-3d	10.524	2.64 %	2.30 %
M-5d	10.261	2.58 %	−0.25 %
M-7d	10.365	2.94 %	0.76 %
H-CONTROL GROUP	15.551	2.65 %	
H-1d	15.236	1.85 %	−2.03 %
H-2d	15.247	1.64 %	−1.95 %
H-3d	15.147	1.95 %	−2.60 %
H-5d	15.248	1.64 %	−1.95 %
H-7d	15.169	1.94 %	−2.46 %
Carbamazepine	Mean value of detection（ug/mL）	CV	
L-CONTROL GROUP	3.125	0.15 %	
L-1d	3.258	0.16 %	4.26 %
L-2d	3.159	0.25 %	1.09 %
L-3d	3.148	0.14 %	0.74 %
L-5d	3.591	0.81 %	14.91 %
L-7d	3.269	0.64 %	4.61 %
M-CONTROL GROUP	10.158	0.55 %	
M-1d	10.264	0.61 %	1.04 %
M-2d	10.258	0.74 %	0.98 %
M-3d	10.264	0.62 %	1.04 %
M-5d	10.267	0.95 %	1.07 %
M-7d	10.543	0.84 %	3.79 %
H-CONTROL GROUP	15.126	0.37 %	
H-1d	15.254	0.65 %	0.85 %
H-2d	15.247	0.82 %	0.80 %
H-3d	15.324	0.31 %	1.31 %
H-5d	15.369	0.61 %	1.61 %
H-7d	15.483	0.51 %	2.36 %
Phenobarbital	Mean value of detection（ug/mL）	CV	
L-CONTROL GROUP	7.416	0.15 %	
L-1d	7.264	0.16 %	−2.05 %
L-2d	7.561	0.07 %	1.96 %
L-3d	7.462	0.04 %	0.62 %
L-5d	7.522	0.28 %	1.43 %
L-7d	7.522	0.41 %	1.43 %
M-CONTROL GROUP	24.69	0.15 %	
M-1d	24.633	0.61 %	−0.23 %
M-2d	24.895	0.51 %	0.83 %
M-3d	24.517	0.95 %	−0.70 %
M-5d	24.366	0.74 %	−1.31 %
M-7d	24.156	0.81 %	−2.16 %
H-CONTROL GROUP	37.811	0.27 %	
H-1d	37.891	0.78 %	0.21 %
H-2d	37.651	1.06 %	−0.42 %
H-3d	37.512	0.15 %	−0.79 %
H-5d	37.652	1.23 %	−0.42 %
H-7d	37.561	0.17 %	−0.66 %
Primidone	Mean value of detection（ug/mL）	CV	
L-CONTROL GROUP	3.891	3.25 %	
L-1d	3.845	3.20 %	−1.18 %
L-2d	3.798	2.05 %	−2.39 %
L-3d	3.658	2.69 %	−5.99 %
L-5d	3.795	2.64 %	−2.47 %
L-7d	3.951	2.81 %	1.54 %
M-CONTROL GROUP	12.546	2.61 %	
M-1d	12.194	2.35 %	−2.81 %
M-2d	12.269	2.34 %	−2.21 %
M-3d	12.514	2.60 %	−0.26 %
M-5d	12.621	2.55 %	0.60 %
M-7d	12.354	2.66 %	−1.53 %
H-CONTROL GROUP	18.245	2.34 %	
H-1d	18.261	2.87 %	0.09 %
H-2d	18.241	2.91 %	−0.02 %
H-3d	18.654	2.54 %	2.24 %
H-5d	18.326	2.68 %	0.44 %
H-7d	18.364	2.15 %	0.65 %
Phenytoin	Mean value of detection（ug/mL）	CV	
L-CONTROL GROUP	3.275	1.23 %	
L-1d	3.548	1.25 %	8.34 %
L-2d	3.375	0.07 %	3.05 %
L-3d	3.287	0.47 %	0.37 %
L-5d	3.269	0.15 %	−0.18 %
L-7d	3.136	0.15 %	−4.24 %
M-CONTROL GROUP	12.136	1.20 %	
M-1d	12.365	1.30 %	1.89 %
M-2d	12.154	1.60 %	0.15 %
M-3d	12.621	1.50 %	4.00 %
M-5d	12.153	1.75 %	0.14 %
M-7d	12.523	2.14 %	3.19 %
H-CONTROL GROUP	18.752	2.30 %	
H-1d	18.504	2.88 %	−1.32 %
H-2d	18.247	2.10 %	−2.69 %
H-3d	18.695	0.78 %	−0.30 %
H-5d	18.16	0.91 %	−3.16 %
H-7d	18.655	0.69 %	−0.52 %
Valproic acid	Mean value of detection（ug/mL）	CV	
L-CONTROL GROUP	18.362	0.84 %	
L-1d	18.256	0.15 %	−0.58 %
L-2d	18.254	0.36 %	−0.59 %
L-3d	18.652	0.24 %	1.58 %
L-5d	18.26	0.51 %	−0.56 %
L-7d	18.456	0.69 %	0.51 %
M-CONTROL GROUP	61.548	0.48 %	
M-1d	61.589	0.15 %	0.07 %
M-2d	61.532	0.67 %	−0.03 %
M-3d	61.587	0.08 %	0.06 %
M-5d	61.52	0.05 %	−0.05 %
M-7d	61.245	0.18 %	−0.49 %
H-CONTROL GROUP	93.278	0.26 %	
H-1d	93.157	0.48 %	−0.13 %
H-2d	93.684	0.69 %	0.44 %
H-3d	93.154	0.15 %	−0.13 %
H-5d	94.012	1.20 %	0.79 %
H-7d	93.158	2.30 %	−0.13 %
Lamotrigine	Mean value of detection（ug/mL）	CV	
L-CONTROL GROUP	3.062	2.64 %	
L-1d	3.058	2.08 %	−0.13 %
L-2d	3.069	2.57 %	0.23 %
L-3d	3.087	2.64 %	0.82 %
L-5d	3.054	2.84 %	−0.26 %
L-7d	3.055	2.51 %	−0.23 %
M-CONTROL GROUP	0.784	0.15 %	
M-1d	0.781	0.45 %	−0.38 %
M-2d	0.782	0.65 %	−0.26 %
M-3d	0.779	0.12 %	−0.64 %
M-5d	0.788	0.61 %	0.51 %
M-7d	0.799	0.54 %	1.91 %
H-CONTROL GROUP	18.854	0.81 %	
H-1d	18.591	0.61 %	−1.39 %
H-2d	18.941	0.51 %	0.46 %
H-3d	18.614	0.64 %	−1.27 %
H-5d	18.251	0.62 %	−3.20 %
H-7d	18.614	0.81 %	−1.27 %
Topiramate	Mean value of detection（ug/mL）	CV	
L-CONTROL GROUP	2.415	0.27 %	
L-1d	2.423	1.50 %	0.33 %
L-2d	2.516	3.90 %	4.18 %
L-3d	2.426	2.47 %	0.46 %
L-5d	2.437	1.34 %	0.91 %
L-7d	2.598	1.19 %	7.58 %
M-CONTROL GROUP	8.125	2.35 %	
M-1d	8.165	1.24 %	0.49 %
M-2d	8.147	1.98 %	0.27 %
M-3d	8.456	1.54 %	4.07 %
M-5d	8.591	1.65 %	5.74 %
M-7d	8.417	1.61 %	3.59 %
H-CONTROL GROUP	12.736	1.45 %	
H-1d	12.845	0.57 %	0.86 %
H-2d	12.651	0.64 %	−0.67 %
H-3d	12.954	0.91 %	1.71 %
H-5d	12.326	0.54 %	−3.22 %
H-7d	12.459	0.87 %	−2.17 %
Levetiracetam	Mean value of detection（ug/mL）	CV	
L-CONTROL GROUP	16.512	5.20 %	
L-1d	16.254	2.61 %	−1.56 %
L-2d	16.231	2.90 %	−1.70 %
L-3d	16.245	3.50 %	−1.62 %
L-5d	16.254	2.10 %	−1.56 %
L-7d	16.325	2.30 %	−1.13 %
M-CONTROL GROUP	48.514	3.15 %	
M-1d	48.651	0.94 %	0.28 %
M-2d	48.572	0.81 %	0.12 %
M-3d	48.136	0.66 %	−0.78 %
M-5d	48.267	0.86 %	−0.51 %
M-7d	48.627	1.25 %	0.23 %
H-CONTROL GROUP	72.215	3.20 %	
H-1d	72.549	2.15 %	0.46 %
H-2d	72.456	2.36 %	0.33 %
H-3d	72.197	3.16 %	−0.02 %
H-5d	72.841	3.26 %	0.87 %
H-7d	72.514	2.58 %	0.41 %
Perampanel	Mean value of detection（ug/mL）	CV	
L-CONTROL GROUP	3.156	1.36 %	
L-1d	3.125	2.36 %	−0.98 %
L-2d	3.254	1.34 %	3.11 %
L-3d	3.169	2.05 %	0.41 %
L-5d	3.158	2.36 %	0.06 %
L-7d	3.265	1.54 %	3.45 %
M-CONTROL GROUP	10.236	1.54 %	
M-1d	10.235	2.58 %	−0.01 %
M-2d	10.951	3.21 %	6.99 %
M-3d	10.542	0.51 %	2.99 %
M-5d	10.354	0.91 %	1.15 %
M-7d	10.254	0.78 %	0.18 %
H-CONTROL GROUP	15.268	0.51 %	
H-1d	15.647	0.34 %	2.48 %
H-2d	15.298	0.35 %	0.20 %
H-3d	15.751	0.16 %	3.16 %
H-5d	15.493	0.16 %	1.47 %
H-7d	15.247	0.45 %	−0.14 %
Zonisamide	Mean value of detection（ug/mL）	CV	
L-CONTROL GROUP	0.012	0.15 %	
L-1d	0.013	0.12 %	8.33 %
L-2d	0.013	0.13 %	8.33 %
L-3d	0.013	4.56 %	8.33 %
L-5d	0.012	0.51 %	0.00 %
L-7d	0.013	0.61 %	8.33 %
M-CONTROL GROUP	0.041	0.41 %	
M-1d	0.042	0.51 %	2.44 %
M-2d	0.041	0.56 %	0.00 %
M-3d	0.043	0.98 %	4.88 %
M-5d	0.041	0.61 %	0.00 %
M-7d	0.045	0.71 %	9.76 %
H-CONTROL GROUP	0.063	0.51 %	
H-1d	0.061	0.84 %	−3.17 %
H-2d	0.062	0.70 %	−1.59 %
H-3d	0.062	0.61 %	−1.59 %
H-5d	0.061	0.62 %	−3.17 %
H-7d	0.063	0.38 %	0.00 %
Clonazepam	Mean value of detection（ug/mL）	CV	
L-CONTROL GROUP	0.156	1.25 %	
L-1d	0.158	1.3.6 %	1.28 %
L-2d	0.148	1.28 %	−5.13 %
L-3d	0.157	2.52 %	0.64 %
L-5d	0.147	1.47 %	−5.77 %
L-7d	0.155	1.35 %	−0.64 %
M-CONTROL GROUP	0.514	1.32 %	
M-1d	0.513	1.64 %	−0.19 %
M-2d	0.519	0.25 %	0.97 %
M-3d	0.518	0.58 %	0.78 %
M-5d	0.517	0.61 %	0.58 %
M-7d	0.511	0.47 %	−0.58 %
H-CONTROL GROUP	0.789	0.26 %	
H-1d	0.784	0.51 %	−0.63 %
H-2d	0.786	0.64 %	−0.38 %
H-3d	0.753	0.14 %	−4.56 %
H-5d	0.791	0.58 %	0.25 %
H-7d	0.779	0.67 %	−1.27 %
9-Hydroxyrisperidone	Mean value of detection（ng/mL）	CV	
L-CONTROL GROUP	4.33	1.33 %	
L-1d	4.17	2.77 %	−3.70 %
L-2d	4.17	1.39 %	−3.70 %
L-3d	4.27	3.58 %	−1.39 %
L-5d	4.23	1.36 %	−2.31 %
L-7d	4.13	1.40 %	−4.62 %
M-CONTROL GROUP	35.53	1.07 %	
M-1d	35.47	1.33 %	−0.17 %
M-2d	34.2	0.00 %	−3.74 %
M-3d	34.07	0.34 %	−4.11 %
M-5d	34.2	0.51 %	−3.74 %
M-7d	34.23	0.34 %	−3.66 %
H-CONTROL GROUP	114.83	0.48 %	
H-1d	113.37	0.75 %	−1.27 %
H-2d	111.87	0.31 %	−2.58 %
H-3d	110.67	0.94 %	−3.62 %
H-5d	114.23	0.39 %	−0.52 %
H-7d	110.03	0.64 %	−4.18 %
Risperidone	Mean value of detection（ng/mL）	CV	
L-CONTROL GROUP	4.77	1.21 %	
L-1d	4.63	1.25 %	−2.94 %
L-2d	4.57	1.26 %	−4.19 %
L-3d	4.73	1.22 %	−0.84 %
L-5d	4.77	1.21 %	0.00 %
L-7d	4.5	0.00 %	−5.66 %
M-CONTROL GROUP	36.6	0.72 %	
M-1d	37	0.81 %	1.09 %
M-2d	35.13	0.43 %	−4.02 %
M-3d	35.33	0.16 %	−3.47 %
M-5d	35.8	1.12 %	−2.19 %
M-7d	35.33	0.16 %	−3.47 %
H-CONTROL GROUP	117.2	0.23 %	
H-1d	116.3	0.73 %	−0.77 %
H-2d	115.03	0.35 %	−1.85 %
H-3d	113.47	0.31 %	−3.18 %
H-5d	117.73	0.40 %	0.45 %
H-7d	115	1.15 %	−1.88 %
N-desmethylolanzapine	Mean value of detection（ng/mL）	CV	
L-CONTROL GROUP	5.6	9.45 %	
L-1d	5.47	7.62 %	−2.32 %
L-2d	5.43	10.78 %	−3.04 %
L-3d	5.23	1.10 %	−6.61 %
L-5d	5.63	1.02 %	0.54 %
L-7d	5.27	8.97 %	−5.89 %
M-CONTROL GROUP	46.27	5.44 %	
M-1d	48.67	4.86 %	5.19 %
M-2d	45.67	5.26 %	−1.30 %
M-3d	44.73	8.41 %	−3.33 %
M-5d	46.7	10.10 %	0.93 %
M-7d	41.67	3.04 %	−9.94 %
H-CONTROL GROUP	144.5	6.50 %	
H-1d	152.13	9.02 %	5.28 %
H-2d	145.4	12.41 %	0.62 %
H-3d	150.5	1.31 %	4.15 %
H-5d	155.9	8.15 %	7.89 %
H-7d	148.17	10.47 %	2.54 %
Olanzapine	Mean value of detection（ng/mL）	CV	
L-CONTROL GROUP	5.7	1.75 %	
L-1d	5.5	0.00 %	−3.51 %
L-2d	5.47	1.06 %	−4.04 %
L-3d	5.57	2.07 %	−2.28 %
L-5d	5.47	1.06 %	−4.04 %
L-7d	5.43	1.06 %	−4.74 %
M-CONTROL GROUP	45.17	0.46 %	
M-1d	45.7	0.22 %	1.17 %
M-2d	43.63	0.48 %	−3.41 %
M-3d	43.3	0.23 %	−4.14 %
M-5d	43.57	1.16 %	−3.54 %
M-7d	43.4	0.69 %	−3.92 %
H-CONTROL GROUP	147.73	0.88 %	
H-1d	145.63	0.88 %	−1.42 %
H-2d	143.7	0.30 %	−2.73 %
H-3d	142.27	0.49 %	−3.70 %
H-5d	147.6	0.98 %	−0.09 %
H-7d	141.57	0.76 %	−4.17 %
N-desmethylclozapine	Mean value of detection（ng/mL）	CV	
L-CONTROL GROUP	36.83	1.50 %	
L-1d	35.83	1.98 %	−2.72 %
L-2d	36.33	1.77 %	−1.36 %
L-3d	37.23	0.41 %	1.09 %
L-5d	36.7	1.52 %	−0.35 %
L-7d	36.97	3.14 %	0.38 %
M-CONTROL GROUP	299.43	1.56 %	
M-1d	298.4	1.63 %	−0.34 %
M-2d	287.33	1.20 %	−4.04 %
M-3d	288.67	2.25 %	−3.59 %
M-5d	291.23	2.00 %	−2.74 %
M-7d	296.83	0.80 %	−0.87 %
H-CONTROL GROUP	962.63	0.29 %	
H-1d	939.2	1.82 %	−2.43 %
H-2d	944.7	0.90 %	−1.86 %
H-3d	926.07	0.89 %	−3.80 %
H-5d	959.83	1.93 %	−0.29 %
H-7d	945.07	2.46 %	−1.82 %
Clozapine	Mean value of detection（ng/mL）	CV	
L-CONTROL GROUP	36.6	0.27 %	
L-1d	35.47	1.72 %	−3.09 %
L-2d	35.7	1.12 %	−2.46 %
L-3d	36.33	1.11 %	−0.74 %
L-5d	36.27	1.41 %	−0.90 %
L-7d	36.33	0.32 %	−0.74 %
M-CONTROL GROUP	301.6	0.95 %	
M-1d	305.4	0.26 %	1.26 %
M-2d	293.63	0.51 %	−2.64 %
M-3d	294.9	1.56 %	−2.22 %
M-5d	300.27	1.32 %	−0.44 %
M-7d	294.83	0.65 %	−2.24 %
H-CONTROL GROUP	976.27	1.34 %	
H-1d	966.57	1.52 %	−0.99 %
H-2d	950.37	0.92 %	−2.65 %
H-3d	960.47	1.07 %	−1.62 %
H-5d	990.7	0.72 %	1.48 %
H-7d	964.27	0.97 %	−1.23 %
Aripiprazole	Mean value of detection（ng/mL）	CV	
L-CONTROL GROUP	35.57	2.03 %	
L-1d	33.3	2.60 %	−6.38 %
L-2d	33.1	4.72 %	−6.94 %
L-3d	33.37	2.11 %	−6.18 %
L-5d	33.83	1.88 %	−4.89 %
L-7d	33.23	1.71 %	−6.58 %
M-CONTROL GROUP	297.63	2.11 %	
M-1d	300.77	2.49 %	1.06 %
M-2d	282.8	2.35 %	−4.98 %
M-3d	284.6	1.35 %	−4.38 %
M-5d	287.1	2.00 %	−3.54 %
M-7d	280.47	1.06 %	−5.77 %
H-CONTROL GROUP	921.3	0.88 %	
H-1d	920.13	1.03 %	−0.13 %
H-2d	899.8	1.56 %	−2.33 %
H-3d	895.97	1.03 %	−2.75 %
H-5d	928.07	1.00 %	0.73 %
H-7d	882.9	0.61 %	−4.17 %
Dehydro aripiprazole	Mean value of detection（ng/mL）	CV	
L-CONTROL GROUP	37.63	0.77 %	
L-1d	35.4	4.18 %	−5.93 %
L-2d	34.8	0.76 %	−7.52 %
L-3d	35.53	1.14 %	−5.58 %
L-5d	35.7	1.22 %	−5.13 %
L-7d	35.07	1.08 %	−6.80 %
M-CONTROL GROUP	311.43	1.08 %	
M-1d	315.9	1.34 %	1.44 %
M-2d	295.23	0.91 %	−5.20 %
M-3d	300.47	1.83 %	−3.52 %
M-5d	300.47	0.19 %	−3.52 %
M-7d	294.53	0.43 %	−5.43 %
H-CONTROL GROUP	929.7	0.83 %	
H-1d	928.87	0.13 %	−0.09 %
H-2d	905.1	0.31 %	−2.65 %
H-3d	903.33	1.11 %	−2.84 %
H-5d	921.17	0.35 %	−0.92 %
H-7d	892.3	1.15 %	−4.02 %
Amisulpride	Mean value of detection（ng/mL）	CV	
L-CONTROL GROUP	22.2	3.90 %	
L-1d	21.77	5.32 %	−1.94 %
L-2d	22.27	5.01 %	0.32 %
L-3d	23.13	2.75 %	4.19 %
L-5d	22.17	1.38 %	−0.14 %
L-7d	22.8	4.39 %	2.70 %
M-CONTROL GROUP	184.4	2.54 %	
M-1d	180.77	2.14 %	−1.97 %
M-2d	177.7	1.50 %	−3.63 %
M-3d	178.2	4.91 %	−3.36 %
M-5d	180.53	5.26 %	−2.10 %
M-7d	190	2.34 %	3.04 %
H-CONTROL GROUP	611.23	3.22 %	
H-1d	588.37	5.11 %	−3.74 %
H-2d	591.17	5.80 %	−3.28 %
H-3d	577.87	0.98 %	−5.46 %
H-5d	603.1	3.62 %	−1.33 %
H-7d	597.33	4.36 %	−2.27 %
Perphenazine	Mean value of detection（ng/mL）	CV	
L-CONTROL GROUP	0.2	0.00 %	
L-1d	0.2	0.00 %	0.00 %
L-2d	0.2	0.00 %	0.00 %
L-3d	0.2	0.00 %	0.00 %
L-5d	0.2	0.00 %	0.00 %
L-7d	0.2	0.00 %	0.00 %
M-CONTROL GROUP	1.6	6.25 %	
M-1d	1.57	3.69 %	−1.88 %
M-2d	1.57	3.69 %	−1.88 %
M-3d	1.5	6.67 %	−6.25 %
M-5d	1.53	3.77 %	−4.38 %
M-7d	1.6	6.25 %	0.00 %
H-CONTROL GROUP	5.4	1.85 %	
H-1d	5.5	3.64 %	1.85 %
H-2d	5.13	1.12 %	−5.00 %
H-3d	5.23	3.98 %	−3.15 %
H-5d	5.57	1.04 %	3.15 %
H-7d	5.37	1.08 %	−0.56 %
Fluphenazine	Mean value of detection（ng/mL）	CV	
L-CONTROL GROUP	0.36	1.08 %	
L-1d	0.34	2.98 %	−5.56 %
L-2d	0.36	0.00 %	0.00 %
L-3d	0.38	3.90 %	5.56 %
L-5d	0.36	2.86 %	0.00 %
L-7d	0.35	1.89 %	−2.78 %
M-CONTROL GROUP	12.63	1.15 %	
M-1d	12.88	2.74 %	1.98 %
M-2d	12.35	1.20 %	−2.22 %
M-3d	12.21	2.92 %	−3.33 %
M-5d	12.31	3.08 %	−2.53 %
M-7d	12.71	1.36 %	0.63 %
H-CONTROL GROUP	24.01	0.81 %	
H-1d	24.05	2.06 %	0.17 %
H-2d	24.11	2.46 %	0.42 %
H-3d	24.16	1.79 %	0.62 %
H-5d	24.38	2.39 %	1.54 %
H-7d	24.17	2.26 %	0.67 %
Haloperidol	Mean value of detection（ng/mL）	CV	
L-CONTROL GROUP	0.5	0.00 %	
L-1d	0.5	0.00 %	0.00 %
L-2d	0.5	0.00 %	0.00 %
L-3d	0.5	0.00 %	0.00 %
L-5d	0.5	0.00 %	0.00 %
L-7d	0.5	0.00 %	0.00 %
M-CONTROL GROUP	4.4	0.00 %	
M-1d	4.4	2.27 %	0.00 %
M-2d	4.27	1.35 %	−2.95 %
M-3d	4.3	2.33 %	−2.27 %
M-5d	4.37	1.32 %	−0.68 %
M-7d	4.37	1.32 %	−0.68 %
H-CONTROL GROUP	14.27	1.07 %	
H-1d	14.03	1.48 %	−1.68 %
H-2d	13.93	1.10 %	−2.38 %
H-3d	13.9	0.72 %	−2.59 %
H-5d	14.6	1.19 %	2.31 %
H-7d	14.13	1.78 %	−0.98 %
Reducd haloperidol	Mean value of detection（ng/mL）	CV	
L-CONTROL GROUP	0.5	4.64 %	
L-1d	0.53	10.83 %	6.00 %
L-2d	0.51	0.00 %	2.00 %
L-3d	0.52	10.67 %	4.00 %
L-5d	0.52	10.19 %	4.00 %
L-7d	0.51	9.12 %	2.00 %
M-CONTROL GROUP	4.8	3.61 %	
M-1d	4.77	2.42 %	−0.63 %
M-2d	4.63	1.25 %	−3.54 %
M-3d	4.77	7.37 %	−0.63 %
M-5d	4.83	1.19 %	0.63 %
M-7d	4.77	3.20 %	−0.63 %
H-CONTROL GROUP	16.33	5.69 %	
H-1d	16.03	2.60 %	−1.84 %
H-2d	15.9	5.76 %	−2.63 %
H-3d	15.93	1.81 %	−2.45 %
H-5d	17.1	4.79 %	4.72 %
H-7d	16.3	3.73 %	−0.18 %
Flupentixol	Mean value of detection（ng/mL）	CV	
L-CONTROL GROUP	0.6	0.00 %	
L-1d	0.6	0.00 %	0.00 %
L-2d	0.63	9.12 %	5.00 %
L-3d	0.6	0.00 %	0.00 %
L-5d	0.6	0.00 %	0.00 %
L-7d	0.6	0.00 %	0.00 %
M-CONTROL GROUP	4.6	2.17 %	
M-1d	4.73	1.22 %	2.83 %
M-2d	4.57	2.53 %	−0.65 %
M-3d	4.5	0.00 %	−2.17 %
M-5d	4.67	1.24 %	1.52 %
M-7d	4.67	5.39 %	1.52 %
H-CONTROL GROUP	15.13	0.38 %	
H-1d	14.9	2.42 %	−1.52 %
H-2d	14.93	2.05 %	−1.32 %
H-3d	14.8	0.68 %	−2.18 %
H-5d	15.27	2.00 %	0.93 %
H-7d	15.17	2.01 %	0.26 %
Quetiapine	Mean value of detection（ng/mL）	CV	
L-CONTROL GROUP	35.43	1.60 %	
L-1d	34.4	0.00 %	−2.91 %
L-2d	34.7	2.18 %	−2.06 %
L-3d	36.03	2.50 %	1.69 %
L-5d	35.07	0.59 %	−1.02 %
L-7d	35.17	1.18 %	−0.73 %
M-CONTROL GROUP	290.7	0.79 %	
M-1d	296.1	1.08 %	1.86 %
M-2d	282.23	1.02 %	−2.91 %
M-3d	285.6	0.40 %	−1.75 %
M-5d	289.1	1.11 %	−0.55 %
M-7d	293.4	0.61 %	0.93 %
H-CONTROL GROUP	961.4	0.48 %	
H-1d	945.97	1.53 %	−1.60 %
H-2d	948.17	1.38 %	−1.38 %
H-3d	934.57	0.46 %	−2.79 %
H-5d	983.63	1.30 %	2.31 %
H-7d	961.33	0.95 %	−0.01 %
Thioridazine	Mean value of detection（ng/mL）	CV	
L-CONTROL GROUP	14.67	0.39 %	
L-1d	14.2	1.41 %	−3.20 %
L-2d	14.23	0.41 %	−3.00 %
L-3d	14.47	0.80 %	−1.36 %
L-5d	14.23	0.81 %	−3.00 %
L-7d	13.97	0.41 %	−4.77 %
M-CONTROL GROUP	124.97	0.12 %	
M-1d	124.8	0.14 %	−0.14 %
M-2d	118.83	0.35 %	−4.91 %
M-3d	117.77	0.30 %	−5.76 %
M-5d	118.17	0.93 %	−5.44 %
M-7d	116.97	0.32 %	−6.40 %
H-CONTROL GROUP	416.57	0.40 %	
H-1d	413.2	0.98 %	−0.81 %
H-2d	404.13	0.61 %	−2.99 %
H-3d	399.47	0.45 %	−4.10 %
H-5d	408.33	0.15 %	−1.98 %
H-7d	393.2	0.23 %	−5.61 %
Lurasidone	Mean value of detection（ng/mL）	CV	
L-CONTROL GROUP	4.23	1.36 %	
L-1d	4.1	0.00 %	−3.07 %
L-2d	4.1	0.00 %	−3.07 %
L-3d	4.2	0.00 %	−0.71 %
L-5d	4.13	1.40 %	−2.36 %
L-7d	4.03	1.43 %	−4.73 %
M-CONTROL GROUP	35.23	0.59 %	
M-1d	35.5	0.56 %	0.77 %
M-2d	34.03	0.17 %	−3.41 %
M-3d	33.8	1.07 %	−4.06 %
M-5d	33.9	0.29 %	−3.78 %
M-7d	33.23	0.17 %	−5.68 %
H-CONTROL GROUP	115.03	0.22 %	
H-1d	113.3	0.55 %	−1.50 %
H-2d	111.37	0.61 %	−3.18 %
H-3d	111.27	1.02 %	−3.27 %
H-5d	114.1	0.84 %	−0.81 %
H-7d	109.27	1.56 %	−5.01 %
Loxapine	Mean value of detection（ng/mL）	CV	
L-CONTROL GROUP	0.7	0.00 %	
L-1d	0.7	0.00 %	0.00 %
L-2d	0.7	0.00 %	0.00 %
L-3d	0.7	0.00 %	0.00 %
L-5d	0.7	0.00 %	0.00 %
L-7d	0.7	0.00 %	0.00 %
M-CONTROL GROUP	6.13	3.39 %	
M-1d	6.2	4.84 %	1.14 %
M-2d	5.73	2.01 %	−6.53 %
M-3d	6.03	2.53 %	−1.63 %
M-5d	6.13	2.49 %	0.00 %
M-7d	5.93	1.95 %	−3.26 %
H-CONTROL GROUP	20.03	1.04 %	
H-1d	19.63	2.30 %	−2.00 %
H-2d	19.3	1.37 %	−3.64 %
H-3d	19.4	2.36 %	−3.15 %
H-5d	20.43	1.98 %	2.00 %
H-7d	19.8	0.51 %	−1.15 %
Chlorpromazine	Mean value of detection（ng/mL）	CV	
L-CONTROL GROUP	20.27	0.57 %	
L-1d	18.67	3.27 %	−7.89 %
L-2d	18.83	0.81 %	−7.10 %
L-3d	19.67	0.29 %	−2.96 %
L-5d	18.7	1.85 %	−7.75 %
L-7d	19.37	1.81 %	−4.44 %
M-CONTROL GROUP	164.83	1.17 %	
M-1d	162.67	1.63 %	−1.31 %
M-2d	153.67	1.26 %	−6.77 %
M-3d	156.07	1.80 %	−5.31 %
M-5d	156.03	3.79 %	−5.34 %
M-7d	156.07	0.46 %	−5.31 %
H-CONTROL GROUP	550.23	2.05 %	
H-1d	504.7	6.23 %	−8.27 %
H-2d	535.93	2.52 %	−2.60 %
H-3d	526.83	1.38 %	−4.25 %
H-5d	527.17	2.84 %	−4.19 %
H-7d	520.73	3.65 %	−5.36 %
Chlorprothixene	Mean value of detection（ng/mL）	CV	
L-CONTROL GROUP	14.77	0.39 %	
L-1d	14.17	1.78 %	−4.06 %
L-2d	14.57	1.05 %	−1.35 %
L-3d	14.97	1.54 %	1.35 %
L-5d	14.9	1.78 %	0.88 %
L-7d	15	1.33 %	1.56 %
M-CONTROL GROUP	125.2	0.32 %	
M-1d	126.53	0.61 %	1.06 %
M-2d	123.57	0.86 %	−1.30 %
M-3d	122	0.50 %	−2.56 %
M-5d	124.67	0.32 %	−0.42 %
M-7d	125.7	1.33 %	0.40 %
H-CONTROL GROUP	385.57	0.58 %	
H-1d	384.5	0.40 %	−0.28 %
H-2d	383.73	0.28 %	−0.48 %
H-3d	383.2	0.28 %	−0.61 %
H-5d	399.37	0.98 %	3.58 %
H-7d	392.37	1.06 %	1.76 %
Ziprasidone	Mean value of detection（ng/mL）	CV	
L-CONTROL GROUP	14.67	3.22 %	
L-1d	13.83	1.67 %	−5.73 %
L-2d	13.83	2.21 %	−5.73 %
L-3d	14	0.71 %	−4.57 %
L-5d	13.7	0.73 %	−6.61 %
L-7d	12.9	0.78 %	−12.07 %
M-CONTROL GROUP	117.07	1.34 %	
M-1d	117.73	0.64 %	0.56 %
M-2d	109.33	0.21 %	−6.61 %
M-3d	109.33	1.05 %	−6.61 %
M-5d	107.47	1.12 %	−8.20 %
M-7d	102.3	0.85 %	−12.62 %
H-CONTROL GROUP	387.47	0.95 %	
H-1d	381.9	2.03 %	−1.44 %
H-2d	371.83	0.51 %	−4.04 %
H-3d	370.07	0.87 %	−4.49 %
H-5d	371.57	1.59 %	−4.10 %
H-7d	344.5	0.95 %	−11.09 %
Sulpiride	Mean value of detection（ng/mL）	CV	
L-CONTROL GROUP	36.03	0.80 %	
L-1d	35.43	1.27 %	−1.67 %
L-2d	35.93	0.85 %	−0.28 %
L-3d	36.93	0.68 %	2.50 %
L-5d	37.2	0.47 %	3.25 %
L-7d	37.17	1.02 %	3.16 %
M-CONTROL GROUP	304	0.32 %	
M-1d	307.43	0.05 %	1.13 %
M-2d	296.7	0.48 %	−2.40 %
M-3d	297.4	0.47 %	−2.17 %
M-5d	303.57	0.54 %	−0.14 %
M-7d	304.1	0.38 %	0.03 %
H-CONTROL GROUP	970.27	0.25 %	
H-1d	970.87	0.26 %	0.06 %
H-2d	958.47	0.65 %	−1.22 %
H-3d	962.5	0.28 %	−0.80 %
H-5d	1005.13	0.08 %	3.59 %
H-7d	987.03	0.49 %	1.73 %
Trazodone	Mean value of detection（ng/mL）	CV	
L-CONTROL GROUP	42.43	0.76 %	
L-1d	41.7	0.72 %	−1.72 %
L-2d	42.17	0.60 %	−0.61 %
L-3d	42.57	1.79 %	0.33 %
L-5d	42.17	0.49 %	−0.61 %
L-7d	41.67	0.37 %	−1.79 %
M-CONTROL GROUP	376.5	0.56 %	
M-1d	380.83	0.17 %	1.15 %
M-2d	365.4	0.68 %	−2.95 %
M-3d	364.37	0.32 %	−3.22 %
M-5d	366.9	0.71 %	−2.55 %
M-7d	363.77	0.48 %	−3.38 %
H-CONTROL GROUP	1165.03	0.92 %	
H-1d	1164.5	1.03 %	−0.05 %
H-2d	1139.07	0.87 %	−2.23 %
H-3d	1134.13	0.54 %	−2.65 %
H-5d	1173.9	0.67 %	0.76 %
H-7d	1132.87	0.59 %	−2.76 %
N-desmetyclomipramine	Mean value of detection（ng/mL）	CV	
L-CONTROL GROUP	15.93	1.31 %	
L-1d	15.67	1.33 %	−1.63 %
L-2d	15.33	1.00 %	−3.77 %
L-3d	16.03	2.00 %	0.63 %
L-5d	15.9	0.63 %	−0.19 %
L-7d	15.93	0.96 %	0.00 %
M-CONTROL GROUP	135.43	0.41 %	
M-1d	135.73	0.38 %	0.22 %
M-2d	131.53	0.53 %	−2.88 %
M-3d	130.03	1.28 %	−3.99 %
M-5d	132.23	1.87 %	−2.36 %
M-7d	132.6	0.93 %	−2.09 %
H-CONTROL GROUP	439.63	0.29 %	
H-1d	437.73	1.15 %	−0.43 %
H-2d	433.3	1.72 %	−1.44 %
H-3d	426.4	0.87 %	−3.01 %
H-5d	449.27	1.09 %	2.19 %
H-7d	435.73	0.58 %	−0.89 %
Clomipramine	Mean value of detection（ng/mL）	CV	
L-CONTROL GROUP	16.3	2.67 %	
L-1d	16.23	1.88 %	−0.43 %
L-2d	16.4	1.22 %	0.61 %
L-3d	17.4	1.00 %	6.75 %
L-5d	16.97	2.45 %	4.11 %
L-7d	17.03	1.22 %	4.48 %
M-CONTROL GROUP	143.4	1.65 %	
M-1d	142.37	1.86 %	−0.72 %
M-2d	140.67	1.33 %	−1.90 %
M-3d	140.27	0.64 %	−2.18 %
M-5d	145.2	0.18 %	1.26 %
M-7d	147.4	1.16 %	2.79 %
H-CONTROL GROUP	449.87	1.88 %	
H-1d	452.9	1.15 %	0.67 %
H-2d	441	1.50 %	−1.97 %
H-3d	443.93	0.34 %	−1.32 %
H-5d	463.5	0.80 %	3.03 %
H-7d	454.03	0.57 %	0.92 %
N-desmethylmianserin	Mean value of detection（ng/mL）	CV	
L-CONTROL GROUP	4.9	2.04 %	
L-1d	4.8	2.08 %	−2.04 %
L-2d	4.87	4.28 %	−0.61 %
L-3d	4.97	1.16 %	1.43 %
L-5d	4.83	1.19 %	−1.43 %
L-7d	4.83	1.19 %	−1.43 %
M-CONTROL GROUP	41.9	0.72 %	
M-1d	42.77	1.20 %	2.08 %
M-2d	40.63	1.02 %	−3.03 %
M-3d	40.5	1.31 %	−3.34 %
M-5d	41.23	1.46 %	−1.60 %
M-7d	41.07	0.51 %	−1.98 %
H-CONTROL GROUP	134.87	1.22 %	
H-1d	133.67	0.87 %	−0.89 %
H-2d	130.9	1.07 %	−2.94 %
H-3d	131	1.60 %	−2.87 %
H-5d	133.3	2.27 %	−1.16 %
H-7d	129.3	1.56 %	−4.13 %
Mianserin	Mean value of detection（ng/mL）	CV	
L-CONTROL GROUP	4.97	3.08 %	
L-1d	4.77	2.42 %	−4.02 %
L-2d	5.07	4.56 %	2.01 %
L-3d	4.97	3.08 %	0.00 %
L-5d	5	2.00 %	0.60 %
L-7d	5.07	6.03 %	2.01 %
M-CONTROL GROUP	41.23	1.88 %	
M-1d	41.77	4.05 %	1.31 %
M-2d	40.5	1.31 %	−1.77 %
M-3d	39.67	1.24 %	−3.78 %
M-5d	40.43	2.30 %	−1.94 %
M-7d	42.13	1.19 %	2.18 %
H-CONTROL GROUP	135.7	1.45 %	
H-1d	130.33	1.31 %	−3.96 %
H-2d	130.43	1.87 %	−3.88 %
H-3d	130.57	3.37 %	−3.78 %
H-5d	135.13	0.66 %	−0.42 %
H-7d	131.63	1.67 %	−3.00 %
N-desmethylmirtazapine	Mean value of detection（ng/mL）	CV	
L-CONTROL GROUP	5.7	0.00 %	
L-1d	5.5	1.82 %	−3.51 %
L-2d	5.5	1.82 %	−3.51 %
L-3d	5.73	1.01 %	0.53 %
L-5d	5.47	1.06 %	−4.04 %
L-7d	5.57	3.74 %	−2.28 %
M-CONTROL GROUP	47.27	0.74 %	
M-1d	47.7	1.17 %	0.91 %
M-2d	45.8	0.66 %	−3.11 %
M-3d	45.53	0.83 %	−3.68 %
M-5d	45.97	2.50 %	−2.75 %
M-7d	45.73	0.91 %	−3.26 %
H-CONTROL GROUP	154.2	0.51 %	
H-1d	152.1	1.26 %	−1.36 %
H-2d	150.37	1.32 %	−2.48 %
H-3d	147.33	0.33 %	−4.46 %
H-5d	152.03	1.26 %	−1.41 %
H-7d	148.73	0.85 %	−3.55 %
Mirtazapine	Mean value of detection（ng/mL）	CV	
L-CONTROL GROUP	5.77	1.00 %	
L-1d	5.7	1.75 %	−1.21 %
L-2d	5.73	1.01 %	−0.69 %
L-3d	5.87	0.98 %	1.73 %
L-5d	5.83	0.99 %	1.04 %
L-7d	5.77	1.00 %	0.00 %
M-CONTROL GROUP	48.27	0.32 %	
M-1d	49.1	0.54 %	1.72 %
M-2d	47.3	0.56 %	−2.01 %
M-3d	47	0.93 %	−2.63 %
M-5d	48.13	1.14 %	−0.29 %
M-7d	47.53	0.74 %	−1.53 %
H-CONTROL GROUP	157.33	0.60 %	
H-1d	157.13	1.53 %	−0.13 %
H-2d	154	0.06 %	−2.12 %
H-3d	154.23	0.29 %	−1.97 %
H-5d	160.27	1.53 %	1.87 %
H-7d	155.77	0.37 %	−0.99 %
N-Desmethylvenlafaxine	Mean value of detection（ng/mL）	CV	
L-CONTROL GROUP	27.77	0.91 %	
L-1d	27.6	0.96 %	−0.61 %
L-2d	27.57	1.05 %	−0.72 %
L-3d	28.33	0.73 %	2.02 %
L-5d	28.13	1.25 %	1.30 %
L-7d	28.17	1.02 %	1.44 %
M-CONTROL GROUP	239.23	0.55 %	
M-1d	242.87	0.64 %	1.52 %
M-2d	235.87	0.29 %	−1.40 %
M-3d	233.07	1.31 %	−2.57 %
M-5d	238.1	0.72 %	−0.47 %
M-7d	237.57	1.28 %	−0.69 %
H-CONTROL GROUP	771.53	0.64 %	
H-1d	769.4	0.54 %	−0.28 %
H-2d	763	0.34 %	−1.11 %
H-3d	755.67	0.67 %	−2.06 %
H-5d	787.43	0.54 %	2.06 %
H-7d	768.8	0.82 %	−0.35 %
O-Desmethylvenlafaxine	Mean value of detection（ng/mL）	CV	
L-CONTROL GROUP	27.4	1.93 %	
L-1d	28.23	1.95 %	3.03 %
L-2d	27.8	2.52 %	1.46 %
L-3d	28.6	2.18 %	4.38 %
L-5d	28.53	0.88 %	4.12 %
L-7d	28.7	1.52 %	4.74 %
M-CONTROL GROUP	245.1	1.80 %	
M-1d	250.4	0.35 %	2.16 %
M-2d	240.97	0.36 %	−1.69 %
M-3d	240.07	1.72 %	−2.05 %
M-5d	244.03	1.72 %	−0.44 %
M-7d	242.27	1.12 %	−1.15 %
H-CONTROL GROUP	787.27	0.96 %	
H-1d	788.8	1.48 %	0.19 %
H-2d	774.8	1.53 %	−1.58 %
H-3d	783.93	0.22 %	−0.42 %
H-5d	812.23	1.30 %	3.17 %
H-7d	789.67	1.45 %	0.30 %
Venlafaxine	Mean value of detection（ng/mL）	CV	
L-CONTROL GROUP	28.37	1.59 %	
L-1d	27.87	2.04 %	−1.76 %
L-2d	28.17	0.20 %	−0.70 %
L-3d	29.1	0.69 %	2.57 %
L-5d	28.63	1.41 %	0.92 %
L-7d	29.07	1.89 %	2.47 %
M-CONTROL GROUP	252.57	0.84 %	
M-1d	252.77	0.70 %	0.08 %
M-2d	246	0.65 %	−2.60 %
M-3d	243.63	0.68 %	−3.54 %
M-5d	247.53	1.82 %	−2.00 %
M-7d	251.53	0.30 %	−0.41 %
H-CONTROL GROUP	769	0.59 %	
H-1d	762.57	0.36 %	−0.84 %
H-2d	756.1	0.26 %	−1.68 %
H-3d	755.47	0.27 %	−1.76 %
H-5d	778.87	0.23 %	1.28 %
H-7d	764.17	1.23 %	−0.63 %
Venlafaxine	Mean value of detection（ng/mL）	CV	
L-CG	28.37	1.59 %	
L-1d	27.87	2.04 %	−1.76 %
L-2d	28.17	0.20 %	−0.70 %
L-3d	29.1	0.69 %	2.57 %
L-5d	28.63	1.41 %	0.92 %
L-7d	29.07	1.89 %	2.47 %
M-CG	252.57	0.84 %	
M-1d	252.77	0.70 %	0.08 %
M-2d	246	0.65 %	−2.60 %
M-3d	243.63	0.68 %	−3.54 %
M-5d	247.53	1.82 %	−2.00 %
M-7d	251.53	0.30 %	−0.41 %
H-CG	769	0.59 %	
H-1d	762.57	0.36 %	−0.84 %
H-2d	756.1	0.26 %	−1.68 %
H-3d	755.47	0.27 %	−1.76 %
H-5d	778.87	0.23 %	1.28 %
H-7d	764.17	1.23 %	−0.63 %
O-Deethylreboxetine	Mean value of detection（ng/mL）	CV	
L-CONTROL GROUP	26.8	2.08 %	
L-1d	25.13	2.33 %	−6.23 %
L-2d	26.3	2.49 %	−1.87 %
L-3d	26.2	0.76 %	−2.24 %
L-5d	26.6	0.75 %	−0.75 %
L-7d	25.63	3.15 %	−4.37 %
M-CONTROL GROUP	238.73	2.68 %	
M-1d	236	2.44 %	−1.14 %
M-2d	229.93	2.03 %	−3.69 %
M-3d	226.7	2.85 %	−5.04 %
M-5d	232.3	1.09 %	−2.69 %
M-7d	227.9	4.25 %	−4.54 %
H-CONTROL GROUP	685.5	1.94 %	
H-1d	641.73	1.18 %	−6.39 %
H-2d	681.53	0.88 %	−0.62 %
H-3d	660.53	4.06 %	−3.66 %
H-5d	693.4	0.29 %	1.20 %
H-7d	693.63	1.26 %	1.17 %
Reboxetine	Mean value of detection（ng/mL）	CV	
L-CONTROL GROUP	25.67	4.76 %	
L-1d	26.2	4.40 %	2.06 %
L-2d	27.03	1.49 %	5.30 %
L-3d	27.33	4.11 %	6.47 %
L-5d	27.47	2.07 %	7.01 %
L-7d	27.9	1.86 %	8.69 %
M-CONTROL GROUP	232.57	1.27 %	
M-1d	241.53	3.12 %	3.85 %
M-2d	239.1	2.98 %	2.81 %
M-3d	237.17	6.80 %	1.98 %
M-5d	238.47	3.52 %	2.54 %
M-7d	235.97	6.31 %	1.46 %
H-CONTROL GROUP	770.57	4.12 %	
H-1d	742	2.94 %	−3.71 %
H-2d	788.57	0.40 %	2.34 %
H-3d	771.33	5.66 %	0.10 %
H-5d	832	8.80 %	7.97 %
H-7d	789.23	2.55 %	2.42 %
Agomelatine	Mean value of detection（ng/mL）	CV	
L-CONTROL GROUP	20.07	1.04 %	
L-1d	19.83	0.77 %	−1.20 %
L-2d	19.67	1.79 %	−1.99 %
L-3d	20.37	1.86 %	1.49 %
L-5d	20.3	2.15 %	1.15 %
L-7d	20.53	1.01 %	2.29 %
M-CONTROL GROUP	173.57	0.37 %	
M-1d	176.33	1.13 %	1.59 %
M-2d	169.47	0.65 %	−2.36 %
M-3d	170.23	1.15 %	−1.92 %
M-5d	172.93	0.95 %	−0.37 %
M-7d	175.63	0.66 %	1.19 %
H-CONTROL GROUP	566.17	0.81 %	
H-1d	564.23	0.83 %	−0.34 %
H-2d	555.13	0.58 %	−1.95 %
H-3d	562.57	1.53 %	−0.64 %
H-5d	585.67	1.00 %	3.44 %
H-7d	573.33	0.56 %	1.26 %
Compound 33: Amitriptyline	Mean value of detection（ng/mL）	CV	
L-CONTROL GROUP	10.93	0.53 %	
L-1d	10.53	1.10 %	−3.66 %
L-2d	10.77	1.93 %	−1.46 %
L-3d	11.1	2.38 %	1.56 %
L-5d	10.93	1.90 %	0.00 %
L-7d	11	0.91 %	0.64 %
M-CONTROL GROUP	90.53	0.89 %	
M-1d	90.8	1.21 %	0.30 %
M-2d	88.93	0.55 %	−1.77 %
M-3d	88.37	1.64 %	−2.39 %
M-5d	89.97	1.70 %	−0.62 %
M-7d	92.23	1.80 %	1.88 %
H-CONTROL GROUP	289.47	1.55 %	
H-1d	288.37	1.11 %	−0.38 %
H-2d	287.23	0.32 %	−0.77 %
H-3d	285.13	0.17 %	−1.50 %
H-5d	298.8	1.22 %	3.22 %
H-7d	291.3	0.84 %	0.63 %
N-Desmethylamitriptyline	Mean value of detection（ng/mL）	CV	
L-CONTROL GROUP	10.77	1.42 %	
L-1d	10.33	2.23 %	−4.09 %
L-2d	10.63	1.44 %	−1.30 %
L-3d	10.7	4.67 %	−0.65 %
L-5d	10.73	1.42 %	−0.37 %
L-7d	10.63	1.96 %	−1.30 %
M-CONTROL GROUP	90.97	1.12 %	
M-1d	91.6	1.44 %	0.69 %
M-2d	89	1.07 %	−2.17 %
M-3d	88.03	2.61 %	−3.23 %
M-5d	89.53	1.59 %	−1.58 %
M-7d	90.73	0.54 %	−0.26 %
H-CONTROL GROUP	294.83	1.43 %	
H-1d	292.97	0.38 %	−0.63 %
H-2d	292.13	0.62 %	−0.92 %
H-3d	290.67	0.87 %	−1.41 %
H-5d	300.63	0.66 %	1.97 %
H-7d	297.7	0.52 %	0.97 %
Bupropion	Mean value of detection（ng/mL）	CV	
L-CONTROL GROUP	71.8	3.29 %	
L-1d	70.67	2.76 %	−1.57 %
L-2d	71.93	1.85 %	0.18 %
L-3d	73.3	1.55 %	2.09 %
L-5d	74	2.71 %	3.06 %
L-7d	74.2	0.88 %	3.34 %
M-CONTROL GROUP	615.47	1.76 %	
M-1d	619.17	0.58 %	0.60 %
M-2d	598.97	0.10 %	−2.68 %
M-3d	605.07	1.62 %	−1.69 %
M-5d	612.27	0.78 %	−0.52 %
M-7d	614.4	0.77 %	−0.17 %
H-CONTROL GROUP	1961.1	0.31 %	
H-1d	1954.07	0.76 %	−0.36 %
H-2d	1940.67	1.09 %	−1.04 %
H-3d	1924.73	0.48 %	−1.85 %
H-5d	2023.23	1.50 %	3.17 %
H-7d	1984.8	0.82 %	1.21 %
Hydroxybupropion	Mean value of detection（ng/mL）	CV	
L-CONTROL GROUP	66.2	2.00 %	
L-1d	63.73	1.60 %	−3.73 %
L-2d	63.67	1.68 %	−3.82 %
L-3d	66.7	0.91 %	0.76 %
L-5d	65.93	1.03 %	−0.41 %
L-7d	68.3	1.25 %	3.17 %
M-CONTROL GROUP	561.87	1.27 %	
M-1d	551.6	1.67 %	−1.83 %
M-2d	535.53	0.31 %	−4.69 %
M-3d	542.3	2.09 %	−3.48 %
M-5d	549.03	2.55 %	−2.29 %
M-7d	556.9	0.68 %	−0.88 %
H-CONTROL GROUP	1917.17	1.28 %	
H-1d	1879.13	2.03 %	−1.98 %
H-2d	1874.93	2.44 %	−2.20 %
H-3d	1844.7	0.93 %	−3.78 %
H-5d	1939.37	1.83 %	1.16 %
H-7d	1914.2	0.95 %	−0.15 %
Duloxetine	Mean value of detection（ng/mL）	CV	
L-CONTROL GROUP	8.8	3.01 %	
L-1d	8.73	8.59 %	−0.80 %
L-2d	8.9	6.83 %	1.14 %
L-3d	8.63	1.34 %	−1.93 %
L-5d	8.73	4.02 %	−0.80 %
L-7d	8.37	11.11 %	−4.89 %
M-CONTROL GROUP	68.97	4.49 %	
M-1d	72.5	1.26 %	5.12 %
M-2d	67	5.60 %	−2.86 %
M-3d	68.4	1.05 %	−0.83 %
M-5d	68.13	1.86 %	−1.22 %
M-7d	69.13	1.84 %	0.23 %
H-CONTROL GROUP	233.83	3.25 %	
H-1d	229.73	2.80 %	−1.75 %
H-2d	232.03	1.81 %	−0.77 %
H-3d	226.4	2.83 %	−3.18 %
H-5d	238.03	0.59 %	1.80 %
H-7d	233.07	2.22 %	−0.33 %
Doxepin	Mean value of detection（ng/mL）	CV	
L-CONTROL GROUP	10.37	3.39 %	
L-1d	10.1	0.99 %	−2.60 %
L-2d	10.37	2.01 %	0.00 %
L-3d	10.4	0.96 %	0.29 %
L-5d	10.47	0.55 %	0.96 %
L-7d	10.57	1.09 %	1.93 %
M-CONTROL GROUP	86.73	0.47 %	
M-1d	88.53	0.57 %	2.08 %
M-2d	84.4	0.43 %	−2.69 %
M-3d	83.5	1.30 %	−3.72 %
M-5d	85.57	0.44 %	−1.34 %
M-7d	86	0.62 %	−0.84 %
H-CONTROL GROUP	287.67	0.56 %	
H-1d	285.47	0.21 %	−0.76 %
H-2d	283.73	1.05 %	−1.37 %
H-3d	281.8	0.44 %	−2.04 %
H-5d	292.77	2.54 %	1.77 %
H-7d	284.87	0.73 %	−0.97 %
N-Desmethyldoxepin	Mean value of detection（ng/mL）	CV	
L-CONTROL GROUP	10.53	2.19 %	
L-1d	10.3	2.91 %	−2.18 %
L-2d	10.33	2.01 %	−1.90 %
L-3d	10.43	1.46 %	−0.95 %
L-5d	10.33	2.01 %	−1.90 %
L-7d	10.43	1.46 %	−0.95 %
M-CONTROL GROUP	90.47	1.57 %	
M-1d	90.9	0.57 %	0.48 %
M-2d	87.73	0.46 %	−3.03 %
M-3d	88.37	1.11 %	−2.32 %
M-5d	89.33	1.39 %	−1.26 %
M-7d	87.5	0.64 %	−3.28 %
H-CONTROL GROUP	289.97	0.33 %	
H-1d	290.53	0.52 %	0.19 %
H-2d	287.3	0.72 %	−0.92 %
H-3d	285.77	0.86 %	−1.45 %
H-5d	296.6	1.03 %	2.29 %
H-7d	288.6	1.03 %	−0.47 %
Vortioxetine	Mean value of detection（ng/mL）	CV	
L-CONTROL GROUP	2.8	0.00 %	
L-1d	2.8	0.00 %	0.00 %
L-2d	2.83	2.04 %	1.07 %
L-3d	2.9	0.00 %	3.57 %
L-5d	2.87	2.01 %	2.50 %
L-7d	2.83	2.04 %	1.07 %
M-CONTROL GROUP	24.03	0.64 %	
M-1d	24.17	0.86 %	0.58 %
M-2d	23.2	0.43 %	−3.45 %
M-3d	23.07	0.25 %	−4.00 %
M-5d	23.53	0.25 %	−2.08 %
M-7d	23.63	0.65 %	−1.66 %
H-CONTROL GROUP	78.03	0.96 %	
H-1d	77.33	0.37 %	−0.90 %
H-2d	75.83	0.66 %	−2.82 %
H-3d	76	0.26 %	−2.60 %
H-5d	79.2	0.77 %	1.50 %
H-7d	77.8	0.13 %	−0.29 %
Fluvoxamine	Mean value of detection（ng/mL）	CV	
L-CONTROL GROUP	17.63	2.29 %	
L-1d	17.03	1.36 %	−3.40 %
L-2d	17.43	2.17 %	−1.13 %
L-3d	17.47	0.87 %	−0.91 %
L-5d	16.87	1.49 %	−4.31 %
L-7d	16.73	0.35 %	−5.10 %
M-CONTROL GROUP	150.87	1.28 %	
M-1d	151.5	0.52 %	0.42 %
M-2d	145.27	1.14 %	−3.71 %
M-3d	143.97	1.26 %	−4.57 %
M-5d	145.23	0.62 %	−3.74 %
M-7d	143.3	1.47 %	−5.02 %
H-CONTROL GROUP	475.43	0.71 %	
H-1d	469.2	0.91 %	−1.31 %
H-2d	460.43	0.70 %	−3.16 %
H-3d	462.9	0.32 %	−2.64 %
H-5d	472.7	0.89 %	−0.57 %
H-7d	456.13	1.06 %	−4.06 %
Fluoxetine	Mean value of detection（ng/mL）	CV	
L-CONTROL GROUP	35.27	5.20 %	
L-1d	33.67	3.12 %	−4.54 %
L-2d	34.73	7.23 %	−1.53 %
L-3d	35.6	3.17 %	0.94 %
L-5d	34.87	3.82 %	−1.13 %
L-7d	35.77	4.07 %	1.42 %
M-CONTROL GROUP	302.83	5.65 %	
M-1d	314.47	3.00 %	3.84 %
M-2d	299.43	1.98 %	−1.12 %
M-3d	295.67	5.04 %	−2.36 %
M-5d	298.53	1.41 %	−1.42 %
M-7d	299.57	1.70 %	−1.08 %
H-CONTROL GROUP	968.37	3.60 %	
H-1d	989.37	2.67 %	2.17 %
H-2d	979.5	4.04 %	1.15 %
H-3d	974.47	2.72 %	0.63 %
H-5d	1016.03	1.95 %	4.92 %
H-7d	983.9	4.13 %	1.60 %
N-Desmethylfluoxetine	Mean value of detection（ng/mL）	CV	
L-CONTROL GROUP	33.57	3.45 %	
L-1d	33	1.99 %	−1.70 %
L-2d	31.53	8.17 %	−6.08 %
L-3d	33.93	6.02 %	1.07 %
L-5d	31.17	0.74 %	−7.15 %
L-7d	33.9	9.43 %	0.98 %
M-CONTROL GROUP	289.27	2.17 %	
M-1d	300.33	1.30 %	3.82 %
M-2d	284.27	1.30 %	−1.73 %
M-3d	281.97	0.32 %	−2.52 %
M-5d	284.77	2.15 %	−1.56 %
M-7d	278.97	2.57 %	−3.56 %
H-CONTROL GROUP	972.33	2.44 %	
H-1d	986.47	2.64 %	1.45 %
H-2d	959.67	2.80 %	−1.30 %
H-3d	930.2	1.23 %	−4.33 %
H-5d	980.53	0.99 %	0.84 %
H-7d	953.17	1.32 %	−1.97 %
Maprotiline	Mean value of detection（ng/mL）	CV	
L-CONTROL GROUP	7.7	3.44 %	
L-1d	7.57	0.76 %	−1.69 %
L-2d	7.73	0.75 %	0.39 %
L-3d	8	1.25 %	3.90 %
L-5d	7.77	1.97 %	0.91 %
L-7d	7.87	1.47 %	2.21 %
M-CONTROL GROUP	65.77	1.67 %	
M-1d	66.53	1.52 %	1.16 %
M-2d	64.43	0.90 %	−2.04 %
M-3d	64.9	1.67 %	−1.32 %
M-5d	65	0.81 %	−1.17 %
M-7d	67.1	1.95 %	2.02 %
H-CONTROL GROUP	217.93	1.72 %	
H-1d	215.2	0.46 %	−1.25 %
H-2d	211.77	0.99 %	−2.83 %
H-3d	213	1.37 %	−2.26 %
H-5d	222.97	2.18 %	2.31 %
H-7d	217.27	1.31 %	−0.30 %
N-Desmethylmaprotiline	Mean value of detection（ng/mL）	CV	
L-CONTROL GROUP	7.9	3.35 %	
L-1d	8.03	3.80 %	1.65 %
L-2d	8.2	5.32 %	3.80 %
L-3d	8.13	1.88 %	2.91 %
L-5d	8.07	3.98 %	2.15 %
L-7d	8.3	2.41 %	5.06 %
M-CONTROL GROUP	67.87	2.15 %	
M-1d	69.57	1.96 %	2.50 %
M-2d	66	1.49 %	−2.76 %
M-3d	65.53	2.34 %	−3.45 %
M-5d	65.97	1.50 %	−2.80 %
M-7d	67.7	0.39 %	−0.25 %
H-CONTROL GROUP	220	0.55 %	
H-1d	220.9	1.85 %	0.41 %
H-2d	214.53	1.22 %	−2.49 %
H-3d	215.87	0.82 %	−1.88 %
H-5d	228.13	1.78 %	3.70 %
H-7d	220.07	1.33 %	0.03 %
Moclobemide	Mean value of detection（ng/mL）	CV	
L-CONTROL GROUP	71	0.51 %	
L-1d	71.67	0.29 %	0.94 %
L-2d	73.67	1.29 %	3.76 %
L-3d	77.13	0.49 %	8.63 %
L-5d	74.32	0.97 %	4.68 %
L-7d	77.02	1.08 %	8.48 %
M-CONTROL GROUP	637.37	0.75 %	
M-1d	655.17	0.50 %	2.79 %
M-2d	647.13	0.67 %	1.53 %
M-3d	656.17	0.89 %	2.95 %
M-5d	683.87	0.60 %	7.30 %
M-7d	705.2	0.58 %	10.64 %
H-CONTROL GROUP	1949.1	0.22 %	
H-1d	1983.67	0.40 %	1.77 %
H-2d	1977.27	0.41 %	1.45 %
H-3d	2005.47	0.24 %	2.89 %
H-5d	2140.37	0.46 %	9.81 %
H-7d	2143.53	0.22 %	9.98 %
Moclobemide N-oxindole	Mean value of detection（ng/mL）	CV	
L-CONTROL GROUP	69.63	0.65 %	
L-1d	66.2	0.40 %	−4.93 %
L-2d	65.17	2.23 %	−6.41 %
L-3d	65.13	1.11 %	−6.46 %
L-5d	60.27	0.25 %	−13.44 %
L-7d	57.97	1.10 %	−16.75 %
M-CONTROL GROUP	619.93	0.02 %	
M-1d	607.83	0.62 %	−1.95 %
M-2d	576.03	1.43 %	−7.08 %
M-3d	555.6	0.80 %	−10.38 %
M-5d	537.77	1.23 %	−13.25 %
M-7d	517.73	1.48 %	−16.49 %
H-CONTROL GROUP	1905.77	1.03 %	
H-1d	1847.2	0.27 %	−3.07 %
H-2d	1802.67	0.82 %	−5.41 %
H-3d	1724.67	1.19 %	−9.50 %
H-5d	1718.57	0.25 %	−9.82 %
H-7d	1592.3	0.58 %	−16.45 %
milnacipran	Mean value of detection（ng/mL）	CV	
L-CONTROL GROUP	10.53	2.19 %	
L-1d	10.5	2.86 %	−0.28 %
L-2d	10.67	3.29 %	1.33 %
L-3d	11.03	1.05 %	4.75 %
L-5d	10.67	1.95 %	1.33 %
L-7d	10.9	1.83 %	3.51 %
M-CONTROL GROUP	91.97	1.31 %	
M-1d	91.57	1.30 %	−0.43 %
M-2d	88.93	1.67 %	−3.31 %
M-3d	90.43	1.99 %	−1.67 %
M-5d	90.4	2.84 %	−1.71 %
M-7d	92.37	0.70 %	0.43 %
H-CONTROL GROUP	292.8	1.03 %	
H-1d	288.37	1.16 %	−1.51 %
H-2d	286.93	1.33 %	−2.00 %
H-3d	286.03	0.33 %	−2.31 %
H-5d	295.4	1.47 %	0.89 %
H-7d	290.4	0.87 %	−0.82 %
Tianeptine	Mean value of detection（ng/mL）	CV	
L-CONTROL GROUP	5.7	1.75 %	
L-1d	5.53	1.04 %	−2.98 %
L-2d	5.53	1.04 %	−2.98 %
L-3d	5.67	1.02 %	−0.53 %
L-5d	5.6	4.72 %	−1.75 %
L-7d	5.67	2.04 %	−0.53 %
M-CONTROL GROUP	49.73	0.81 %	
M-1d	50.33	0.83 %	1.21 %
M-2d	48.13	1.02 %	−3.22 %
M-3d	48.77	2.02 %	−1.93 %
M-5d	48.83	2.26 %	−1.81 %
M-7d	48.4	1.24 %	−2.67 %
H-CONTROL GROUP	154.77	2.51 %	
H-1d	152.4	3.62 %	−1.53 %
H-2d	155.6	1.22 %	0.54 %
H-3d	151.87	1.75 %	−1.87 %
H-5d	156.33	3.30 %	1.01 %
H-7d	153.67	2.60 %	−0.71 %
Paroxetine	Mean value of detection（ng/mL）	CV	
L-CONTROL GROUP	4.13	1.40 %	
L-1d	4.1	0.00 %	−0.73 %
L-2d	4.1	2.44 %	−0.73 %
L-3d	4.27	1.35 %	3.39 %
L-5d	4.17	1.39 %	0.97 %
L-7d	4.17	1.39 %	0.97 %
M-CONTROL GROUP	35.7	1.22 %	
M-1d	36.13	0.16 %	1.20 %
M-2d	34.47	0.93 %	−3.45 %
M-3d	34.17	1.03 %	−4.29 %
M-5d	34.7	0.86 %	−2.80 %
M-7d	35.1	0.99 %	−1.68 %
H-CONTROL GROUP	116.67	0.60 %	
H-1d	114.53	2.49 %	−1.83 %
H-2d	112.4	1.18 %	−3.66 %
H-3d	111.37	0.76 %	−4.54 %
H-5d	116.73	1.12 %	0.05 %
H-7d	114.23	1.28 %	−2.09 %
Norsertraline	Mean value of detection（ng/mL）	CV	
L-CONTROL GROUP	11.03	0.52 %	
L-1d	10.2	2.59 %	−7.52 %
L-2d	10.77	3.75 %	−2.36 %
L-3d	10.8	8.83 %	−2.09 %
L-5d	10.67	3.01 %	−3.26 %
L-7d	10.9	3.18 %	−1.18 %
M-CONTROL GROUP	94.47	0.82 %	
M-1d	96.1	2.09 %	1.73 %
M-2d	93.1	0.67 %	−1.45 %
M-3d	93.47	0.86 %	−1.06 %
M-5d	94.67	0.78 %	0.21 %
M-7d	96.27	0.33 %	1.91 %
H-CONTROL GROUP	303.13	1.85 %	
H-1d	298.37	0.40 %	−1.57 %
H-2d	293.97	0.84 %	−3.02 %
H-3d	296.63	1.89 %	−2.14 %
H-5d	308.83	1.01 %	1.88 %
H-7d	310.27	0.39 %	2.36 %
Compound 54: Sertraline	Mean value of detection（ng/mL）	CV	
L-CONTROL GROUP	10.7	0.93 %	
L-1d	10.33	2.44 %	−3.46 %
L-2d	10.43	2.00 %	−2.52 %
L-3d	10.9	2.43 %	1.87 %
L-5d	10.9	0.92 %	1.87 %
L-7d	10.97	2.63 %	2.52 %
M-CONTROL GROUP	89.7	2.06 %	
M-1d	90.53	1.64 %	0.93 %
M-2d	88.2	0.41 %	−1.67 %
M-3d	88.23	0.29 %	−1.64 %
M-5d	89.6	0.67 %	−0.11 %
M-7d	90.8	0.48 %	1.23 %
H-CONTROL GROUP	288.63	0.43 %	
H-1d	288.63	1.31 %	0.00 %
H-2d	284.93	1.64 %	−1.28 %
H-3d	285.6	0.46 %	−1.05 %
H-5d	301.37	1.39 %	4.41 %
H-7d	295.93	0.34 %	2.53 %
Citalopram	Mean value of detection（ng/mL）	CV	
L-CONTROL GROUP	7.6	2.28 %	
L-1d	7.6	1.32 %	0.00 %
L-2d	7.6	2.28 %	0.00 %
L-3d	7.8	1.28 %	2.63 %
L-5d	7.93	0.73 %	4.34 %
L-7d	7.83	0.74 %	3.03 %
M-CONTROL GROUP	66.53	0.71 %	
M-1d	66.57	1.62 %	0.06 %
M-2d	64.47	0.50 %	−3.10 %
M-3d	64.9	0.56 %	−2.45 %
M-5d	65.87	0.61 %	−0.99 %
M-7d	66.93	1.83 %	0.60 %
H-CONTROL GROUP	211.8	0.90 %	
H-1d	208.87	1.15 %	−1.38 %
H-2d	206.33	1.21 %	−2.58 %
H-3d	206.37	0.83 %	−2.56 %
H-5d	214.13	0.26 %	1.10 %
H-7d	211.6	1.23 %	−0.09 %
N-Desmethylcitalopram	Mean value of detection（ng/mL）	CV	
L-CONTROL GROUP	5.33	1.08 %	
L-1d	5.13	2.98 %	−3.75 %
L-2d	5.1	0.00 %	−4.32 %
L-3d	5.33	3.90 %	0.00 %
L-5d	5.33	2.86 %	0.00 %
L-7d	5.3	1.89 %	−0.56 %
M-CONTROL GROUP	43.4	1.15 %	
M-1d	42.33	2.74 %	−2.47 %
M-2d	41.87	1.20 %	−3.53 %
M-3d	41.7	2.92 %	−3.92 %
M-5d	42.87	3.08 %	−1.22 %
M-7d	43.07	1.36 %	−0.76 %
H-CONTROL GROUP	150.4	0.81 %	
H-1d	149.07	2.06 %	−0.88 %
H-2d	145.87	2.46 %	−3.01 %
H-3d	144.27	1.79 %	−4.08 %
H-5d	151.13	2.39 %	0.49 %
H-7d	149.23	2.26 %	−0.78 %

### 3.6 Carryover effect

The residues in the blank samples after the high concentration samples of each compound were analyzed. The residues of the LLOQ were 0.00 %–18.73 % and the residues of the internal standard were 0.00 %–0.77 %, meeting the requirements(Table 7).

**Table 7:** Carryover evaluations of 71 neuropsychotropic drugs.

Compound	signal response	residuals
Oxcarbazepine		
STD1	67008.008	
BLANK	147.346	0.22 %
internal standard	178438.984	
BLANK	28.332	0.02 %
10-hydroxycarbazepine	signal response	residuals
STD1	31815.13	
BLANK	1359.354	4.27 %
internal standard	574373.4377	
BLANK	254.823	0.04 %
Carbamazepine epoxide	signal response	residuals
STD1	5053.924	
BLANK	5.574	0.11 %
internal standard	19524.076	
BLANK	23.321	0.12 %
Carbamazepine	signal response	residuals
STD1	32546.385	
BLANK	44.881	0.14 %
internal standard	171859.672	
BLANK	7.56	0.00 %
Phenobarbital	signal response	residuals
STD1	350.979	
BLANK	1.509	0.43 %
internal standard	2556.573	
BLANK	0.416	0.02 %
Primidone	signal response	residuals
STD1	1698.904	
BLANK	5.297	0.31 %
internal standard	6158.382	
BLANK	3.003	0.05 %
Phenytoin	signal response	residuals
STD1	49084.25	
BLANK	809.863	1.65 %
internal standard	678922.6253	
BLANK	638.129	0.09 %
Valproic acid	signal response	residuals
STD1	28940.939	
BLANK	682.318	2.36 %
internal standard	375832.5	
BLANK	2903.159	0.77 %
Lamotrigine	signal response	residuals
STD1	3462.948	
BLANK	5.999	0.17 %
internal standard	185723.594	
BLANK	59.937	0.03 %
Topiramate	signal response	residuals
STD1	4336.462	
BLANK	0.072	0.00 %
internal standard	51674.66	
BLANK	2.723	0.01 %
Levetiracetam	signal response	residuals
STD1	887.625	
BLANK	4.956	0.56 %
internal standard	12817.592	
BLANK	6.541	0.05 %
Perampanel	signal response	residuals
STD1	12045.884	
BLANK	125.83	1.04 %
internal standard	46777.227	
BLANK	4.343	0.01 %
Zonisamide	signal response	residuals
STD1	53068.25	
BLANK	41.556	0.08 %
internal standard	460376.906	
BLANK	18.081	0.00 %
Clonazepam	signal response	residuals
STD1	967.745	
BLANK	2.384	0.25 %
internal standard	4943.215	
BLANK	0.46	0.01 %
9-Hydroxyrisperidone	signal response	residuals
STD1	7519.58	
BLANK	402.292	5.35 %
internal standard	240959.1097	
BLANK	69.828	0.03 %
Risperidon	signal response	residuals
STD1	13621.26	
BLANK	1142.98	8.39 %
internal standard	342820.4583	
BLANK	254.398	0.07 %
N-demethylolanzapine	signal response	residuals
STD1	5204.69	
BLANK	68.557	1.32 %
internal standard	70367.961	
BLANK	68.174	0.10 %
Olanzapine	signal response	residuals
STD1	18941.85	
BLANK	557.7	2.94 %
internal standard	300507.875	
BLANK	122.592	0.04 %
N-demethylclozapine	signal response	residuals
STD1	29973.94	
BLANK	1649.867	5.50 %
internal standard	484209.7813	
BLANK	205.971	0.04 %
Clozapine	signal response	residuals
STD1	15981.72	
BLANK	550.617	3.45 %
internal standard	641428.8753	
BLANK	206.601	0.03 %
Aripiprazole	signal response	residuals
STD1	17723.21	
BLANK	3319.531	18.73 %
internal standard	252106	
BLANK	789.311	0.31 %
Dehydro aripiprazole	signal response	residuals
STD1	14185.08	
BLANK	2511.892	17.71 %
internal standard	358991.2293	
BLANK	1660.119	0.46 %
Amisulpride	signal response	residuals
STD1	8143.83	
BLANK	2.835	0.03 %
internal standard	141477.8333	
BLANK	18.715	0.01 %
Perphenazine	signal response	residuals
STD1	623.18	
BLANK	70.734	11.35 %
internal standard	4765.851	
BLANK	11.038	0.23 %
Fluphenazine	signal response	residuals
STD1	1513.65	
BLANK	192.346	12.71 %
internal standard	38917.28533	
BLANK	31.015	0.08 %
Haloperidol	signal response	residuals
STD1	2599.44	
BLANK	258.448	9.94 %
internal standard	43956.67833	
BLANK	5.084	0.01 %
Reduced haloperidol	signal response	residuals
STD1	1300.27	
BLANK	72.771	5.60 %
internal standard	25923.78933	
BLANK	39.771	0.15 %
Flupentixol	signal response	residuals
STD1	452.94	
BLANK	39.399	8.70 %
internal standard	5335.198	
BLANK	24.833	0.47 %
Quetiapine	signal response	residuals
STD1	20758.24	
BLANK	39.151	0.19 %
internal standard	409608.2707	
BLANK	67.407	0.02 %
Thioridazine	signal response	residuals
STD1	30436.60	
BLANK	3266.496	10.73 %
internal standard	527893.792	
BLANK	783.946	0.15 %
Lurasidone	signal response	residuals
STD1	15117.99	
BLANK	1948.518	12.89 %
internal standard	208296.3337	
BLANK	377.159	0.18 %
Loxapine	signal response	residuals
STD1	857.53	
BLANK	24.846	2.90 %
internal standard	17347.11133	
BLANK	0	0.00 %
Chlorpromazine	signal response	residuals
STD1	17006.77	
BLANK	1615.134	9.50 %
internal standard	381348.5	
BLANK	376.167	0.10 %
Chlorprothixene	signal response	residuals
STD1	11729.59	
BLANK	1219.897	10.40 %
internal standard	319217.3543	
BLANK	254.589	0.08 %
Ziprasidone	signal response	residuals
STD1	14042.39	
BLANK	1836.155	13.08 %
internal standard	1282090.875	
BLANK	2986.791	0.23 %
Sulpiride	signal response	residuals
STD1	15213.23	
BLANK	63.104	0.41 %
internal standard	440069.417	
BLANK	46.86	0.01 %
Trazodone	signal response	residuals
STD1	32756.95	
BLANK	1255.644	3.83 %
internal standard	567631.3753	
BLANK	219.696	0.04 %
N-desmetyclomipramine	signal response	residuals
STD1	4085.09	
BLANK	204.182	5.00 %
internal standard	91246.11967	
BLANK	379.106	0.42 %
Clomipramine	signal response	residuals
STD1	49615.51	
BLANK	1595.538	3.22 %
internal standard	827137.2713	
BLANK	573.154	0.07 %
N-desmethylmianserin	signal response	residuals
STD1	9229.72	
BLANK	277.453	3.01 %
internal standard	100681.065	
BLANK	198.783	0.20 %
Mianserin	signal response	residuals
STD1	5627.82	
BLANK	49.248	0.88 %
internal standard	100681.065	
BLANK	198.783	0.20 %
N-desmethylmirtazapine	signal response	residuals
STD1	24472.11	
BLANK	798.558	3.26 %
internal standard	561924.542	
BLANK	267.182	0.05 %
Mirtazapine	signal response	residuals
STD1	10704.44	
BLANK	268.827	2.51 %
internal standard	226143.1873	
BLANK	66.171	0.03 %
N-desmethylvenlafaxine	signal response	residuals
STD1	31454.40	
BLANK	1021.896	3.25 %
internal standard	516892.9897	
BLANK	0	0.00 %
O-desmethylvenlafaxine	signal response	residuals
STD1	31592.59	
BLANK	58.524	0.19 %
internal standard	593638.3543	
BLANK	7.119	0.00 %
Venlafaxine	signal response	residuals
STD1	10751.96	
BLANK	380.881	3.54 %
internal standard	414906.1563	
BLANK	62.669	0.02 %
O-Deethylreboxetine	signal response	residuals
STD1	56809.70	
BLANK	1305.888	2.30 %
internal standard	1271758.25	
BLANK	186.83	0.01 %
Reboxetine	signal response	residuals
STD1	45725.84	
BLANK	1135.442	2.48 %
internal standard	1271758.25	
BLANK	186.83	0.01 %
Agomelatine	signal response	residuals
STD1	43436.97	
BLANK	1163.809	2.68 %
internal standard	703808.5003	
BLANK	188.105	0.03 %
Amitriptyline	signal response	residuals
STD1	17836.85	
BLANK	882.473	4.95 %
internal standard	310194.0003	
BLANK	389.802	0.13 %
N-Desmethylamitriptyline	signal response	residuals
STD1	12824.42	
BLANK	346.121	2.70 %
internal standard	213636.229	
BLANK	292.975	0.14 %
Bupropion	signal response	residuals
STD1	3474.19	
BLANK	222.879	6.42 %
internal standard	314383.042	
BLANK	185.352	0.06 %
Hydroxybupropion	signal response	residuals
STD1	57362.51	
BLANK	1056.073	1.84 %
internal standard	678922.6253	
BLANK	638.129	0.09 %
Duloxetine	signal response	residuals
STD1	193.75	
BLANK	9.036	4.66 %
internal standard	8807.121	
BLANK	0	0.00 %
Doxepin	signal response	residuals
STD1	25206.07	
BLANK	775.263	3.08 %
internal standard	356734	
BLANK	211.755	0.06 %
N-Desmethyldoxepin	signal response	residuals
STD1	22331.01	
BLANK	714.696	3.20 %
internal standard	554384.792	
BLANK	368.927	0.07 %
Vortioxetine	signal response	residuals
STD1	11607.49	
BLANK	564.594	4.86 %
internal standard	579110.438	
BLANK	519.709	0.09 %
Fluvoxamine	signal response	residuals
STD1	24072.38	
BLANK	954.102	3.96 %
internal standard	1441909.125	
BLANK	446.77	0.03 %
Fluoxetine	signal response	residuals
STD1	10313.14	
BLANK	109.261	1.06 %
internal standard	206301.0937	
BLANK	24.35	0.01 %
N-Desmethylfluoxetine	signal response	residuals
STD1	2198.76	
BLANK	78.01	3.55 %
internal standard	78848.44267	
BLANK	14.973	0.02 %
Maprotiline	signal response	residuals
STD1	17853.64	
BLANK	550.795	3.09 %
internal standard	277760.4063	
BLANK	186.812	0.07 %
N-Desmethylmaprotiline	signal response	residuals
STD1	2440.68	
BLANK	39.397	1.61 %
internal standard	277760.4063	
BLANK	186.812	0.07 %
Moclobemide	signal response	residuals
STD1	57547.66	
BLANK	569.417	0.99 %
internal standard	817380.667	
BLANK	64.462	0.01 %
Moclobemide-N-Oxide	signal response	residuals
STD1	18163.92	
BLANK	330.903	1.82 %
internal standard	674666.3127	
BLANK	97.981	0.01 %
milnacipran	signal response	residuals
STD1	21987.32	
BLANK	465.463	2.12 %
internal standard	438519.3233	
BLANK	88.924	0.02 %
Tianeptine	signal response	residuals
STD1	17317.57	
BLANK	427.805	2.47 %
internal standard	263915.5107	
BLANK	33.705	0.01 %
Paroxetine	signal response	residuals
STD1	5517.43	
BLANK	271.286	4.92 %
internal standard	106850.8907	
BLANK	119.59	0.11 %
Norsertraline	signal response	residuals
STD1	14901.40	
BLANK	486.313	3.26 %
internal standard	298408.6253	
BLANK	156.883	0.05 %
Sertraline	signal response	residuals
STD1	9065.38	
BLANK	424.018	4.68 %
internal standard	138939.6197	
BLANK	62.085	0.04 %
Citalopram	signal response	residuals
STD1	24704.09	
BLANK	1232.026	4.99 %
internal standard	520500.9897	
BLANK	107.653	0.02 %
N-Desmethylcitalopram	signal response	residuals
STD1	10250.44	
BLANK	464.705	4.53 %
internal standard	235076.2607	
BLANK	10.788	0.00 %

## 4 Discussion

Regional surveys in recent years have shown that the monitoring of psychotropic drug concentrations in China is still in its infancy, and the low coverage of inter-room QC and the lack of a unified detection method are unmet needs [24,25]. Kirchherr et al. established a method for the simultaneous detection of 48 psychiatric drugs, but their method employed only 3 isotopic internal standards, which did not correspond to all compounds, and the detection time of this method was relatively long [26]. Two reported methods by Remane et al. and Montenarh et al. were able to detect more than 30 psychiatric drugs simultaneously, but pre-treatment for both methods required complex liquid-liquid extraction [27,28]. We hereby propose a method that is better than the reported ones in terms of number of compounds detected and sample pretreatment.

In this study, a UPLC-MS/MS detection method was established for the simultaneous determination of 71 neuropsychotropic drugs. During optimization of the method, it was found that the negative ion mode detection signals of phenobarbital, phenytoin, and valproic acid were higher and stabler than those obtained in the positive ion mode. Therefore, ammonium acetate was added to the mobile phase to improve the negative ion response, while taking into account that the vast majority of the drugs need to be detected in the positive ion mode. Acetic acid was also added to the mobile phase, and the concentrations of acetic acid and ammonium acetate were optimized for better compatibility of the signals with the two detection modes.

Our proposed method avoided the solvent effect by using methanol-acetonitrile as the protein precipitant, performing centrifugation at low temperature and high speed to separate the drugs to be tested, and adding methanol-water before sampling. The method was found to be simple and easy to perform and could meet the requirements of high-throughput TDM. The linear range of each drug covered the therapeutic concentration range provided by the AGNP guidelines and the laboratory alert concentration. All compounds except moclobemide N-indole were stable for 7 days at room temperature, but degradation of olanzapine, oxcarbazepine, bupropion, and other drugs stored at −80 °C for 30 days was noted. However, the degradation could be avoided by the addition of 5 % 200 mg/mL vitamin C (final concentration of vitamin C was 10 mg/mL) as an antioxidant. In this case, the stability result of bupropion was in accordance with the AGNP guideline, and the stability result of olanzapine was inconsistent with the reported data [[29], [30], [31]]. The stability of 10-hydroxycarbazepine is inconsistent with some studies, possibly because the sample used in our study was spiked serum rather than patient serum. When using UPLC-MS/MS for quantitative analysis, the ideal internal standard is the isotopic marker of the component to be measured. Accordingly, we used the respective isotopic markers of the drugs as the internal standard, so that the internal standard peak coincide with the analyte peak to reduce the matrix effect on quantification [32]. However, there is one shortcoming: the matrix effect, we only evaluated one serum and this method did not fully assess the matrix effect. Another advantage of proposed method is that it requires a small sample volume of only 50 μL, which opens up the possibility of detecting samples from neonatal and pediatric populations and also reduces reagent costs.

## 5 Conclusion

The UPLC-MS/MS method proposed could successfully allow the simultaneous determination of serum drug concentrations of 71 clinical neuropsychotropic drugs with excellent sensitivity and specificity. These properties are essential for the accurate identification and quantification of a wide range of neuropsychotropic drugs in the serum, ensuring that the method can reliably discriminate between compounds without cross-reactivity. In addition, the precision, accuracy, and stability of the method met the requirements of the relevant guidelines. Methanol-acetonitrile was used to precipitate proteins in this study. This method is very simple and easy to implement in a laboratory setting. In addition, the method requires a low sample volume of only 50 μL of serum. One of the most notable features of the method is the ability to obtain results rapidly. Each sample could be analyzed in less than 6 min, making our method significantly faster than many previously reported methods. A rapid turnaround is critical for timely clinical decision making, especially in cases of drug intoxication or clinical drug adjustments [[33], [34], [35]].

## CRediT authorship contribution statement

**Weifeng Jin:** Writing – review & editing, Writing – original draft, Visualization, Validation. **Jianhua Wang:** Methodology. **Shuzi Chen:** Investigation. **Qing Chen:** Project administration. **Dan Li:** Investigation. **Mengyuan Zhu:** Data curation. **Xiaomei Fu:** Investigation. **Yingyu Huang:** Methodology. **Ping Lin:** Investigation, Funding acquisition, Conceptualization.

## Declaration of competing interest

We have no known competing financial interests or personal relationships that could have appeared to influence the work reported in this paper.
